# Condensin I is required for faithful meiosis in Drosophila males

**DOI:** 10.1007/s00412-020-00733-w

**Published:** 2020-04-08

**Authors:** Kristina Kleinschnitz, Nina Vießmann, Mareike Jordan, Stefan K. Heidmann

**Affiliations:** 1 Lehrstuhl für Genetik, University of Bayreuth, Bayreuth, Germany; 2 Max Planck Institute of Molecular Cell Biology and Genetics, Dresden, Germany

**Keywords:** Condensin, Meiosis, Drosophila, CRISPR/Cas9

## Abstract

The heteropentameric condensin complexes play vital roles in the formation and faithful segregation of mitotic chromosomes in eukaryotes. While the different contributions of the two common condensin complexes, condensin I and condensin II, to chromosome morphology and behavior in mitosis have been thoroughly investigated, much less is known about the specific roles of the two complexes during meiotic divisions. In *Drosophila melanogaster*, faithful mitotic divisions depend on functional condensin I, but not on condensin II. However, meiotic divisions in Drosophila males require functional condensin II subunits. The role of condensin I during male meiosis in Drosophila has been unresolved. Here, we show that condensin I-specific subunits localize to meiotic chromatin in both meiosis I and II during Drosophila spermatogenesis. Live cell imaging reveals defects during meiotic divisions after RNAi-mediated knockdown of condensin I-specific mRNAs. This phenotype correlates with reduced male fertility and an increase in nondisjunction events both in meiosis I and meiosis II. Consistently, a reduction in male fertility was also observed after proteasome-mediated degradation of the condensin I subunit Barren. Taken together, our results demonstrate an essential role of condensin I during male meiosis in *Drosophila melanogaster*.

**Electronic supplementary material:**

The online version of this article (10.1007/s00412-020-00733-w) contains supplementary material, which is available to authorized users.

## Introduction

Faithful distribution of the replicated genetic information onto the daughter cells is critically dependent on chromosome condensation, which represents the transformation of the dispersed interphase chromatin into rod-like and sturdy metaphase chromosomes. The essential participation of the heteropentameric condensin complexes in this process has been thoroughly demonstrated (for review see (Hirano 2016; Hudson et al. 2009; Kschonsak and Haering 2015; Piskadlo and Oliveira 2016; Takahashi and Hirota 2019; Wood et al. 2010). Mitotic chromosomes isolated from condensin-depleted cells are much more sensitive towards chemical or mechanical stress when compared to chromosomes from mock-depleted cells (Hudson et al. 2003; Ono et al. 2017; Sun et al. 2018). Also, condensin is clearly essential for compaction of sperm chromatin incubated in *Xenopus laevis* egg extracts (Hirano et al. 1997; Hirano and Mitchison 1994), and it is one of the six purified components required for in vitro reconstitution of mitotic chromatids (Shintomi et al. 2017; Shintomi et al. 2015). However, in many systems, the phenotypes which are observed after condensin depletion affect the structure of mitotic chromosomes only slightly. The extent of the compaction phenotype varies by the organism studied and the experimental system used (for review see (Hirano 2012). In most cases, a distinct phenotype can be observed in anaphase, which is referred to as anaphase bridges. These structures represent persistent interconnections of chromatin fibers, resulting in severe problems during chromatid segregation in late mitosis. Thus, in addition to compaction of mitotic chromatin, condensin also has a crucial role in ensuring proper resolution of chromatin entanglements, most likely in concert with topoisomerase II (Piskadlo and Oliveira 2017). Since anaphase bridges are much more prominent after condensin depletion than condensation phenotypes, the former process appears to be more sensitive towards reduced condensin levels. The lack of clear condensation phenotypes in many cases could be attributed to incomplete depletion of the respective condensin subunits (Hirano 2016).

Metazoans harbor two condensin complexes, both containing the structural maintenance of chromosomes (SMC) proteins SMC2 and SMC4, but differing in their non-SMC regulatory subunits. Condensin I complexes contain the subunits Cap-D2, Cap-G, and Cap-H (also called Barren in Drosophila; we will use Barren for the Drosophila Cap-H homolog throughout), while condensin II complexes contain the related subunits Cap-D3, Cap-G2, and Cap-H2. Cap-H and Cap-H2 belong to the kleisin family of proteins, which are characterized by their ability to bind to the head/neck domains of SMC protein dimers (Onn et al. 2007; Schleiffer et al. 2003). Cap-G, Cap-G2, Cap-D2, and Cap-D3 contain in their N-terminal parts extended regions of Huntingtin, elongation factor 3, A-subunit of protein phosphatase 2A, TOR1 lipid kinase (HEAT) repeats which have been shown to mediate protein-protein interactions (Andrade et al. 2001; Hara et al. 2019). In vertebrates, both condensin complexes play essential roles and collaborate in structuring of mitotic chromosomes and in ensuring their unperturbed segregation. The two complexes fulfill non-overlapping functions as exemplified by distinct phenotypes upon depletion of either condensin I or condensin II-specific subunits (Gerlich et al. 2006; Green et al. 2012; Hirota et al. 2004), or by their spatially alternating association with mitotic chromosomes (Ono et al. 2004; Ono et al. 2003). In fact, condensin II was shown to be the primary complex required for establishing a proper mitotic chromosome architecture in human cells (Ono et al. 2017). In Xenopus egg extracts, reconstitution experiments have shown that condensin I is primarily responsible for axial compaction and condensin II for longitudinal compaction of mitotic chromatin (Shintomi and Hirano 2011). Furthermore, the two complexes are differentially localized in interphase: Condensin I-specific subunits are enriched in the cytoplasm, while condensin II-specific subunits can be found primarily in the nucleus (Gerlich et al. 2006; Hirota et al. 2004; Ono et al. 2004). Mechanistically, condensin complexes organize mitotic chromatin into an ordered array of loops sized ~ 450 kb (condensin II) and ~ 80 kb (condensin I) catalyzed by ATP-dependent loop extrusion with one strand of DNA actively transported through the ring-shaped structure formed by the SMC and the kleisin subunits (Ganji et al. 2018; Gibcus et al. 2018; Naumova et al. 2013; Walther et al. 2018).

The clear-cut functional specialization of the two condensin complexes is not strictly conserved, because the occurrence and the composition of the condensin complexes found in different eukaryotes is not uniform. Fission and budding yeast, as well as ciliates and kinetoplastids, harbor homologs only for condensin I (for review see Hirano 2012; Howard-Till and Loidl 2018). On the other hand, *C. elegans* contains three condensin complexes, one of which (condensin I^DC^) has specialized to function in dosage compensation in hermaphrodites (Csankovszki et al. 2009). In *Drosophila melanogaster*, condensin I is present, but for condensin II only the specific subunits Cap-H2 and Cap-D3 have been identified. Despite thorough genetic and biochemical analyses, no Cap-G2 subunit could be found in the fly (Herzog et al. 2013). In fact, a recent query of insect genomes has revealed that species in many taxa lack one or more condensin II-specific genes (King et al. 2019). Obviously, in the taxa without a complete condensin II set of proteins, this complex may have evolved to perform different tasks besides organization of mitotic chromosomes. This notion is clearly supported by the fact that loss-of-function mutations of the Drosophila genes encoding Cap-H2 and Cap-D3 are viable, indicating that their function is dispensable for mitotic proliferation (Hartl et al. 2008a; Hartl et al. 2008b; Savvidou et al. 2005). However, Cap-H2 and Cap-D3 mutant males are sterile, and cytological as well as genetic evidence clearly indicates a role during male meiosis for these two subunits (Hartl et al. 2008b). It has also been shown that Drosophila Cap-H2 negatively regulates chromosome associations, and genetic evidence indicates that this function is dependent on Cap-D3 (Hartl et al. 2008a). Moreover, Cap-D3 has been shown to influence innate immunity in collaboration with Rbf (Longworth et al. 2008; Longworth et al. 2012), to restrict retrotransposon mobilization (Schuster et al. 2013) and to regulate EGFR activity in the developing wing (Klebanow et al. 2016). Thus, the Drosophila condensin II subunits Cap-H2 and Cap-D3 perform multiple roles, including regulation of gene expression, as has been demonstrated for both condensin complexes in other studies (Cobbe et al. 2006; Dej et al. 2004; Gosling et al. 2007; Xu et al. 2006).

While a great amount of work has been devoted to unraveling the functions of condensin in mitosis, the role of condensin in meiosis is only beginning to unfold. In budding yeast, condensin has been shown to participate in repair of double-strand breaks, chromosome axis morphogenesis, recombinatorial repair, resolution of recombination-dependent linkages between homologs, as well as homolog co-orientation in meiosis I by cooperating with the monopolin complex (Brito et al. 2010; Yu and Koshland 2005; Yu and Koshland 2003). In *C. elegans*, condensins have been shown to influence meiotic DNA double-strand break distribution due to their effect on higher-order chromosome structure (Mets and Meyer 2009). Also, *C. elegans* condensin I protects cohesion complexes from premature release from meiotic chromatin by Wapl. Consequently, pairing and synapsis defects occur, when condensin I is impaired (Hernandez et al. 2018). In mouse oocytes, antibody injection experiments revealed the importance of both condensin complexes for establishing and maintaining a condensed structure of the chromosomes but also a role in the mono-orientation of synapsed homologs during meiosis I (Lee et al. 2011). However, targeted inactivation of condensin complexes during oocyte development in mice demonstrated a major contribution of condensin II for the establishment of the structure of meiotic chromatin (Houlard et al. 2015), while condensin I appears to be of minor importance. In Drosophila, *Cap-H2* and *Cap-D3* mutant males exhibit phenotypes already during the extended prophase of meiosis I. Typically, the bivalents of the major autosomes and the gonosomes are organized in distinct and well separated so-called chromosome territories juxtaposed to the nuclear membrane of spermatocytes. These territories are largely absent in *Cap-H2* and *Cap-D3* mutant males, and the chromatin appears dispersed throughout the nucleoplasm. Furthermore, the meiotic divisions observed in *Cap-H2* and *Cap-D3* mutant males are characterized by the occurrence of anaphase bridges, pointing towards a disturbed chromatin structure in male meiosis (Hartl et al. 2008b). While *Cap-H2* mutants also show a distinctive phenotype during oogenesis (failure to disassemble polytene nurse cell chromosomes), a role of condensin II-specific subunits in female meiotic processes has not been established (Hartl et al. 2008a). On the other hand, certain female sterile *Cap-G* alleles result in defective metaphase I figures in oocytes, suggesting a role for condensin I in structuring chromatin during female meiosis (Resnick et al. 2009). Thus, it is conceivable that in Drosophila, the two complexes have specialized with condensin I being more important in female meiosis, while condensin II is the major determinant in male meiosis. However, studies assessing an involvement of any of the condensin I-specific subunits in structuring chromatin during male meiosis in Drosophila have not been published.

We show here that condensin I subunits colocalize with chromatin during meiosis in Drosophila males. RNAi-mediated depletion of condensin I leads to reduced fertility coupled with high levels of chromosome nondisjunction in both meiotic divisions. Cytological analyses in males with reduced condensin I function reveal anaphase bridges in meiosis I and II, and a high proportion of aneuploid gametes. These effects can be recapitulated by targeted proteasomal degradation of the condensin I specific subunit Barren. Thus, our studies demonstrate the importance of condensin I for faithful meiotic divisions in Drosophila.

## Materials and methods

### Drosophila stocks

Fly stocks were obtained from the Bloomington *Drosophila* Stock Center (BDSC) at Indiana University, unless indicated otherwise. Flies were kept on standard fly food at 25 °C.

To analyze chromatin loading, we used fly strains coexpressing EGFP-tagged condensin subunits and His2Av-mRFP1. Lines for the analysis of SMC2 (w*; *P [w*^*+*^, *His2Av-mRFP1]II.1*; *M [w*^*+*^, *gSMC2*_*h*_*-EGFP]ZH-96E*) and Cap-D2 (w*; *M [w*^*+*^, *EGFP-Cap-D2]ZH-51D/CyO*; *P [w*^*+*^, *His2Av-mRFP1]III.1/TM3*,*Sb*) were described previously (Herzog et al. 2013). Lines expressing C-terminally fused variants of Barren and Cap-G were generated using a CRISPR/Cas9-based approach (see below). The resulting Cap-G-FE and Barren-FE transgenes were then combined with a chromosome carrying a transgene allowing expression of His2Av fused with mRFP1 (Schuh et al. 2007).

For RNAi-mediated knockdown of Condensin mRNA, we used fly strains expressing either short hairpin RNAs (shRNAs), as in the case of SMC2 (*y*^*1*^sc* v^*1*^; *P [TRiP.HMS00360]*attP2) and Cap-G (w*; *P [w*^*+*^, *UAS-CapG-RNAi 20.2]ZH-96E*), or a long hairpin RNA (lhRNA) as in the case of Barren (P [KK101679]VIE-260B). The Cap-G shRNA expressing construct was generated by cloning a double-stranded oligonucleotide corresponding to nucleotides 1148-1168 of the Cap-G reading frame, flanked by NheI and EcoRI sites, into the plasmid pWalium20, which is identical to pValium20 (Ni et al. 2011) except that it contains *white*^*+*^ as selectable marker instead of *vermilion*^+^. The construct was injected into *y*^*1*^, *w*^*1*^, *M [vas-int]ZH2A*; *M[3xP3-RFP*, *attP’]ZH96E* embryos to establish transgenic lines.

For expression of UAS-transgenes, we used *bam-GAL4-VP16* (Chen and McKearin 2003), *ey-GAL4* (Hazelett et al. 1998), and maternal *α4tub-GAL4-VP16* (Micklem et al. 1997).

For the construction of fly stocks expressing siRNA-resistant variants, siRNA target sites of *SMC2* (CAAAACAAGTTCCTCATCAA) and *Cap-G* (GGCAGTGTCTTAGCGAATATC) were mutated based on a PCR-mediated approach. A first fragment comprising the *SMC2* genomic sequence up to the region encoding the mutated siRNA target site was PCR-amplified using the primers KS39 (5′-GCGGTTAATTAAACGTTAAAATAATTGAATGAAGC-3′) and KS42 (5′-CCATTAATCAGAAATTTATTTTTGCCTCCGACAACCAC-3′). A second fragment corresponding to a region spanning the mutated target site and the downstream sequence of the target site was PCR-amplified using the primers KS40 (5′-ATAAACGCGTATGACGCAGCTCGATCTCTGAGGTC-3′) and KS41 (5′-GGCAAAAATAAATTTCTGATTAATGGCAAGCTGGTGC-3′). The two PCR-generated DNA fragments partially overlap in the region encoding the mutated siRNA recognition site. After purification (PCR purification kit, Thermo Scientific), the two PCR products were pooled and served as template for a final PCR using the flanking primers KS39 and KS40. The final PCR product was used to replace the native sequence in the plasmid *pattB-SMC2*_*h*_*-EGFP* (Herzog et al. 2013). Target site mutation of *Cap-G* was carried out analogous to that described above for *SMC2* but with primer pairs IH01 (5′-ATATCCTAGGGGCTGAGGAGGGCAATGAG-3′)/IH02 (5′-CCAGGTACTCGGACAGGCATTGCCAATATAACAACAGC-3′), IH03 (5′-ATTGGCAATGCCTGTCCGAGTACCTGGAGACGGAAGCG-3′)/IH04 (5′-ATCACTAAGTGAAAGTTAATTAAGTTAG-3′), and IH01/IH04 for the final amplification. As template, the plasmid *pattB-Cap-G*^*FL*^*-EGFP* (Herzog et al. 2013) was used. For target site mutation of *Barren*, 104 silent mutations were introduced in the 530 bp spanning recognition site by gene synthesis and this region was replaced within the plasmid *pattB-barren-EGFP*. *pattB-barren-EGFP* contains a 6.8 kb genomic fragment encompassing *barren* as well as 1247 bp and 3219 bp of genomic sequences upstream and downstream of the *barren* reading frame, respectively. The *EGFP* reading frame was fused to the 3′-terminus of the *barren* reading frame. This genomic *barren-EGFP* transgene fully rescues the lethality associated with the *barr*^*L305*^*/Df(2 L)Exel7077* transheterozygous mutant situation (data not shown). Transgenic lines of the siRNA-resistant transgenes were generated by φC31 integrase-mediated germline transformation via injection of the plasmids *pattB-Cap-G-siRNA*^*res*^*, pattB-SMC2*_*h*_*-siRNA*^*res*^, and *pattB-barren-siRNA*^*res*^ into *y*^*1*^w* M [vas-int. Dm]ZH-2A; M[3xP3-RFP.attP’]ZH-68E embryos*.*

For deGradFP dependent destruction of Barren-FE in the male germ line, we generated w*; *Barren-FE/Barren-FE*; *P [w*^*+*^,*UASP-NSlmb-vhh-GFP4]III.1/ P [w*^*+*^*, bam-GAL4-VP16]* or w*; *Barren-FE/CyO*; *P [w*^*+*^,*UASP-NSlmb-vhh-GFP4]III.1/ P [w*^*+*^, *bam-GAL4-VP16]* males by standard crossing schemes. As controls, we also generated w*; *Barren-FE/Barren-FE*; *P [w*^*+*^,*UASP-NSlmb-vhh-GFP4]III.1/TM3*, *Sb* or w*; *Barren-FE/CyO*; *P [w*^*+*^,*UASP-NSlmb-vhh-GFP4]III.1/ TM3, Sb* flies, which do not carry a Gal4 driver. The w*; *P [w*^*+*^, *UASP-NSlmb-vhh-GFP4]III.1* transgene was described previously (Urban et al. 2014).

### CRISPR/Cas9-mediated genome engineering

#### gRNA design

To generate variants of Barren and Cap-G carrying a C-terminal EGFP-fusion expressed from the endogenous loci, we employed the CRISPR/Cas9-induced HDR pathway to insert the coding sequence for EGFP downstream of their respective reading frames within the genome. Target sites for Cas9 were chosen in a way that the double-strand breaks (DSBs) were generated in close proximity to the designated fusion site, i.e., the translational termination codon, and with a low risk of potential off-target effects. To identify optimal target sequences and assess specificity of the CRISPR targets, we used the CRISPR Optimal Target Finder algorithm at http://tools.flycrispr.molbio.wisc.edu/targetFinder/ (Gratz et al. 2014). In order to supply gRNAs from a plasmid DNA source, designated target site sequences (*Barren*: 5′-GCTAATTCCGCAGGAGGACTTGG-3′ ➔ cleavage 36 nt upstream of the translational stop codon within exon 3 of *Barren*; *Cap-G*: 5′-GAAGCGCGTGACGCGGGCAGTGG-3′ ➔ cleavage 48 nt upstream of the translational stop codon within exon 6 of *Cap-G*) were synthesized as a pair of short complementary oligonucleotides and cloned into the pU6-BbsI-gRNA vector backbone (Gratz et al. 2014) according to the instructions provided on *flyCRISPR*.

#### HDR template cloning

The HDR templates were assembled in the plasmid pSLfa1180fa (Horn and Wimmer 2000) and contained the EGFP-encoding sequence preceded by a removable FRT-SV40-3xP3-FRT (or initially FRT-3xP3-FRT) expression cassette. These regions were flanked by appropriate homologous sequences (at least 1 kb homology arms upstream and downstream of the cleavage site) for efficient HDR-mediated repair. To avoid cleavage of the HDR templates by Cas9, silent point mutations were introduced into the protospacer regions and the PAM sites. The details of the cloning strategy are available upon request.

#### Microinjection and screening

HDR templates and the gRNA-encoding plasmids were co-injected into transgenic embryos expressing Cas9 under control of the *nos*-promotor (Port et al. 2014). Six micrograms of gRNA- and 6 μg HDR template-encoding plasmids were co-precipitated and dissolved in 20 μl of injection buffer (0.1 mM NaP, 5 mM KCl; pH 6.8) prior to injection. To isolate integration events, individual adult males developing from injected embryos were outcrossed to females of the balancer stock w*; *Sco/CyO*, *P [ry*^*+*^, *ftz lacZ]*. Each F1 brood was scored for green eye fluorescence due to expression of EGFP under control of the eye-specific 3xP3-promotor, indicating integration events. Individual recombined chromosomes were isolated and the resulting balanced lines are referred to as *Barren-FSV3FE/CyO*, *P [ry*^*+*^, *ftz lacZ]* or w*; *Cap-G-FSV3FE/CyO*, *P [ry*^*+*^, *ftz lacZ]* or w*; *Cap-G-F3FE/CyO*, *P [ry*^*+*^, *ftz lacZ]* (the latter lacking the SV40 terminator sequence). The correct integration at the desired locus was confirmed by PCR analyses.

To remove the promotor-cassette, and at the same time generate a translational fusion between Barren or Cap-G and EGFP, Flp mRNA was injected into embryos derived from parents with the genotypes w*; *Barren-FSV3FE/CyO*, *P [ry*^*+*^, *ftz lacZ]* or w*; *Cap-G-FSV3FE/CyO*, *P [ry*^*+*^, *ftz lacZ]* or w*; *Cap-G-F3FE/CyO*, *P [ry*^*+*^, *ftz lacZ]*. Single-injected, non-CyO males were outcrossed to w*; *Sco/CyO*, *P [ry*^*+*^, *ftz lacZ]* virgin females and the F1 generation was screened for the loss of green eye fluorescence.

#### Flp mRNA production

The Flp-recombinase encoding sequence was amplified with the oligodeoxynucleotides SH381 (5′-CGATCATAATACGACTCACTATAGGGGTCACAACATGGGCCCAAAAAAGAAAAGA-3′) and SH382 (5′-ATGGCGCGCCTTATATGCGTC-3′) from the plasmid pAS1834 (generously provided by Olaf Stemmann). The PCR product served as template for Flp mRNA synthesis by in vitro transcription and subsequent polyadenylation using the *mMessage mMachine R T7 Ultra Kit* (Thermo Fisher, Invitrogen) according to the manufacturers’ recommendations.

#### Culture of isolated spermatid cysts

To determine the chromatin association of the EGFP-fused condensin subunits during male meiosis, pupal testes were dissected at approximately 1 day after puparium formation in Shields and Sang M3 insect culture medium (Sigma) supplemented with 10% fetal bovine serum (Sigma) as well as 100 U/ml penicillin and 100 μg/ml streptomycin. The dissected pupal testes were transferred into culture dishes (ibidi μ-Dish; article no. 81158) and teared open with thin needles. Spermatid cysts were gently squeezed out of the testes. For microscopy, isolated cysts were transferred into sterile glass-bottom culture dishes (ibidi 8-well μ-Slide; article no. 80826) with fresh medium using glass Pasteur pipettes. To avoid floating of the cysts, a drop of 1% methyl cellulose was added to each well.

Cysts were staged according to their morphology and the presence of three chromatin territories indicating prophase of meiosis I. For analysis of progression through meiosis II, appropriate cysts were identified by the size and the number of the nuclei within the cysts. Multi-stack confocal images were acquired every 3 min using a Leica Confocal TCS SP5 system (Carl Zeiss,Germany) equipped with a × 40/1.25 oil-immersion objective, a 488-nm Ar laser, and a 561-nm DPSS561 laser for the excitation of EGFP and mRFP1, respectively.

#### Male fertility test

To assess male fertility, 2–4-day-old single males were crossed to three 5–12-day-old virgin wild-type females. Ten single males were analyzed per genotype. Crosses were maintained at 25 °C on standard medium supplemented with yeast paste. After 4 days, males were discarded and females were flipped into fresh vials and maintained for a second period of 4 days. Females were then removed and all vials were further incubated at 25 °C. Progeny was counted over a period of 10 days starting with the first day of eclosion. For statistical analysis, unpaired Student’s *t* tests (www.graphpad.com) were performed.

#### Analysis of seminal vesicles and early embryos

To analyze sperm content within seminal vesicles, males were collected shortly after eclosion, restricted from females and maintained at 25 °C. After 10 days, seminal vesicles were dissected and fixed at room temperature for 20 min in a mixture of 300 μl heptane and 150 μl fixation solution (1× PBS, 0.5% Nonidet NP 40 and 2% para-formaldehyde). Fixed seminal vesicles were then washed twice with PBS and treated with Hoechst 33258 (1 μg/ml in PBS) to stain DNA. Confocal microscopy was employed to determine the focal plane showing the maximum extent of the seminal vesicles. ImageJ (Schneider et al. 2012) was used to calculate the area of the seminal vesicles within these focal planes.

For analysis of early embryonic development, 0–3-h-old embryos were collected on apple-juice agar plates and dechorionized. Embryos were fixed in a 1:1 mixture of n-heptane and methanol for 5 min at room temperature, washed with PBST (PBS plus 0.1% Triton X-100) and treated with Hoechst 33258 (1 μg/ml in PBS) to stain DNA. Prior to mounting, embryos were washed three times in PBS for 5 min each.

#### Nondisjunction analysis

For the analysis of nondisjunction rates of the 4th chromosome after knockdown of condensin subunits in the male germ line, we adopted and modified a previously published assay (Hartl et al. 2008b). Twenty males (2–3 days old) were crossed to 30 virgin females carrying the compound chromosome *C(4) RM, ci*^*1*^*, ey*^*R*^. Because in these females the 4th chromosomes are attached, the eggs either carry the compound *C(4) RM*, *ci ey* chromosome (diplo-4), or no 4th chromosome (nullo-4). Nullo-4 eggs fertilized by normal haploid sperm create nullo-4/+ progeny, while the fertilization of *C(4) RM*, *ci*^*1*^, *ey*^*R*^ eggs with haploid sperm creates *C(4) RM*, *ci*^*1*^, *ey*^*R*^/+ progeny. Both classes are viable and appear normal with respect to *ci* and *ey* according to wild-type alleles on the paternal 4th chromosome. In the case of 4th chromosome missegregation events during male meiosis, exceptional classes of progeny arise, one of which can be phenotypically detected. This is, when nullo-4 sperm fertilize diplo-4 oocytes. In this case, progeny exhibit the *ci* and *ey* mutant phenotype due to carrying exclusively the mutant alleles present on the compound chromosome, which are not complemented by wild type alleles provided by the father. The additional exceptional classes go undetected with this assay because they are either lethal (*0/0*) or appear phenotypically wild-type (*0/++* and *C(4) RM*, *ci*^*1*^, *ey*^*R*^/*++*).

To quantify sex chromosome nondisjunction rates, 20 males (2–3 days old) were crossed to 30 virgin wild-type females. In addition to the transgenes needed for downregulation of condensin subunits, males were bearing an Y chromosome (*Dp(1*;*Y)B*^*s*^*Yy*^*+*^) carrying two X translocations with a dominant allele of *Bar (B*^*S*^*)* and the wild-type allele of *yellow*, respectively. The *y*^*+*^ allele was dispensable for the evaluation of the nondisjunction test. Offspring that arises from sperm bearing the normal sex chromosome content, either one X or one Y, corresponds to the genotypes XX and X *B*^*s*^*Yy*^*+*^, respectively. In this case, all female flies have a wild-type eye phenotype, whereas all males have reduced eyes due to the *B*^*S*^ allele. If exceptional classes of sperm are created that are XY, or lack either sex chromosome entirely, then B^S^ females (XX *B*^*s*^*Yy*^*+*^) and males with wild-type eyes (X0 males) arise, respectively, among the offspring. Diplo-X sperm will result in a lethal Triplo-X combination after fertilization.

To specifically analyze X chromosome nondisjunction during meiosis II, 20 males (2–3 days old) were crossed to 30 virgin females of the genotype *C(1) RM*, *y*^*2*^*su (w*^*a*^*)*^*1*^*w*^*a*^*/0* (BDSC stock no. 700) carrying a compound X-chromosome. Regular female progeny from these crosses inherits the compound X chromosome from the mothers and a Y chromosome from the fathers. These females are phenotypically characterized by wild-type eye color and yellow pigmentation of the cuticle. If X-chromosome nondisjunction occurs during meiosis II in the fathers, sperm containing two X-chromosomes can fertilize eggs without a gonosome resulting in female progeny that can be phenotypically distinguished from their siblings. These exceptional females are *yellow*^*+*^, and carry a *w*^*−*^ -allele on their X-chromosomes from the father. In the case of Cap-G-RNAi, all exceptional progeny harbor one *mini-white*^*+*^ allele due to the presence of either the *bam-GAL4* or the *UAS-Cap-G-RNAi* transgene, which result in orange eyes. In the case of Barren-RNAi the progeny receives none, one, or two copies of a *mini-white*^*+*^ marked transgene, since *bam-GAL4* and *UAS-Barren-RNAi* reside on different chromosomes. Thus, the progeny has white, orange or red eyes, respectively.

#### Squashed testes preparations and immunofluorescence staining

3–4 pairs of testes were dissected from young males of the desired genotype and placed into a drop of PBS (137 mM NaCl; 2.7 mM KCl; 10 mM Na_2_HPO_4_; 1.8 mM KH_2_PO_4_; pH 7.4) on a poly-L-lysine-coated microscopy slide. A siliconized cover slip was placed on the samples, covered with 4 layers of tissue, and testes were gently squashed by applying some pressure manually. After snap-freezing in liquid nitrogen, the cover slip was immediately removed using a clean scalpel. The slides were then transferred into a Coplin jar filled with ice-cold 95% ethanol, and dehydrated at − 20 °C for at least 10 min. Samples were treated with 4% formaldehyde in PBS for 10 min at room temperature to fix the testes. After fixation, slides were washed two times with PBST (PBS/0.3% Triton X-100) and once with PBT (PBS/0.1% Tween 20) 15 min each. For the blocking step, slides were immersed in PBT/1% BSA for 30 min. Slides were removed from the jar; 60 μl of primary antibody solution was applied to the squashed testes, protected with a cover slip, and incubated in a dark, moist chamber at 4 °C overnight. After washing four times in PBT/1% BSA for 15 min each, secondary goat antibodies conjugated with Alexa 488 or Cy3 were applied for 2 h analogous to the primary antibody treatment. All antibodies were diluted in PBT/1% BSA. Following additional washes with PBT/1% BSA, DNA was stained with Hoechst 33258 (1 μg/ml). Finally, samples were washed four times in PBS and mounted with Fluoromount G (Southern Biotech).

#### Fluorescent in situ hybridization (FISH)

A labeled X-chromosome-specific probe was prepared and used in FISH analyses as described previously (Urban et al. 2014) with some modifications. Testes were dissected from 1 to 2-day old males and incubated in a droplet of 0.5% sodium citrate on a microscopy slide coated with poly-L-lysine for 10 min. The sodium citrate solution was carefully removed, and testes were coated with a 45% acetic acid/2% para-formaldehyde solution for 3 min to fix the sample. Following a squashing step as described above, testes were dehydrated sequentially with ice-cold 70% and 100% ethanol. After air-drying, testes were sequentially incubated with 2× SSCT (0.3 M sodium chloride; 30 mM sodium citrate; 0.1% (*v*/v) Tween 20), 2× SSCT-25% formamide, and 2× SSCT-50% formamide for 10 min each, followed by incubation in fresh 2× SSCT-50% formamide for 3 h at 37 °C. The testes were then coated with 36 μl of hybridization buffer (20% dextrane sulfate, 15% formamide in 2× SSCT) supplemented with 100 ng of fluorescently labeled probe and protected with a cover slip. Probe and chromosomal DNA were denatured at 95 °C for 5 min, and the hybridization reaction was carried out overnight at 37 °C in a humid chamber. After hybridization, slides were washed three times with pre-warmed (37 °C) 2× SSCT-50% formamide for 1 h each, then once with 2× SSCT-25% formamide, and once with 2× SSCT for 10 min/wash. The samples were rinsed with PBS and DNA was stained with Hoechst 33258 (1 μg/ml in PBS). Finally, the testes were washed once in PBS and mounted in Fluoromount G Medium (Southern Biotech).

#### Immunoblotting

For immunoblotting experiments, tissues were dissected in PBS and homogenized in sodium dodecyl sulfate-polyacrylamide gel electrophoresis (SDS-PAGE) sample buffer. Protein samples were then separated on Tris-glycine-based polyacrylamide gels and blotted onto nitrocellulose membranes. For detection of bound antibodies on immunoblots, the horseradish peroxidase-based system from p.j.k was used according to the manufacturer’s recommendations.

#### Antibodies

Antibodies against Drosophila SMC2, Drosophila Cap-G, Drosophila Cid, and EGFP have been described previously (Herzog et al. 2013; Jäger et al. 2005). An antibody against Barren was raised in rabbits using bacterially expressed full-length protein as antigen. The antiserum was affinity purified using standard procedures. A mouse monoclonal antibody directed against α-tubulin was obtained from Sigma. For immunoblotting, rabbit antibodies were used at a 1∶3000 dilution and the anti-α-tubulin antibody at a 1:20,000 dilution. For immunofluorescence analyses, the anti-Cid antibody and the anti-α-tubulin antibody were diluted 1:500 and 1:8000, respectively. Secondary antibodies conjugated with fluorophores (Molecular probes) or horseradish peroxidase (Jackson laboratories) were obtained commercially.

## Results

### CRISPR/Cas9-generated EGFP-fusions of Cap-G and Barren

**Fig. 1:**
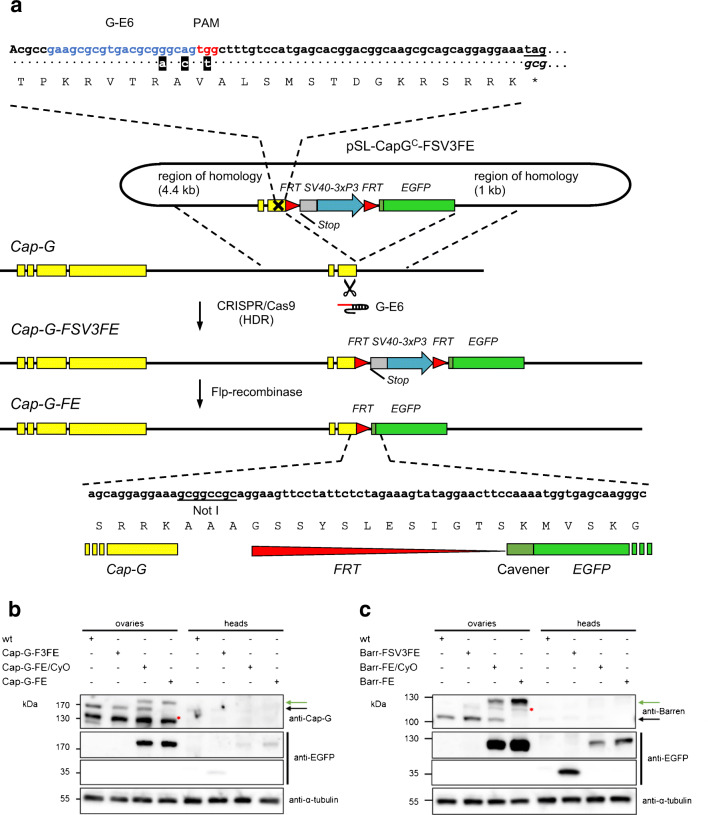
Strategy for the C-terminal tagging of endogenous loci with EGFP via CRISPR/Cas9. **a** Schematic illustration of the strategy using the example of Cap-G. The gRNA G-E6 targets a site 48 nucleotides upstream of the *Cap-G* translational stop codon within exon 6. Nucleotides in blue represent the guide sequence of the gRNA, and nucleotides in red the PAM-sequence. Below the genomic sequence stretch of the 3′-terminal region of Cap-G, the alterations within the HDR template are illustrated. Nucleotides shown in white on black denote silent mutations that were introduced into the HDR template to prevent its targeting by the gRNA. Nucleotides in italics represent the first three nucleotides of the FRT site replacing the Cap-G translational stop codon. Upper case letters indicate the deduced amino acid sequence of the Cap-G C-terminus. pSL-CapG^C^-FSV3FE is the template used for homology directed repair. It contains 4.4 kb of the genomic Cap-G region fused to a FRT site (red arrowhead) in frame, immediately upstream of the translational termination codon (Stop). The adjacent SV40 terminator region (gray box), is followed by the 3xP3 promoter region (blue arrow) a second FRT site, the EGFP coding region (green box), and 1 kb of genomic region downstream of the Cap-G translational stop codon. The EGFP coding sequence is preceded by the Cavener consensus sequence (CAAA) optimizing translation initiation (dark green box). The X within the coding region of exon 6 illustrates the three closely spaced point mutations preventing targeting by G-E6. After cleavage of the genomic locus by Cas9 associated with G-E6, HDR leads to insertion of the FRT-SV40-3xP3-FRT-EGFP cassette (FSV3FE). The resulting transgenic flies express EGFP under control of the eye-specific promoter 3xP3. Ensuing Flp-recombinase-mediated excision of the SV40-3xP3-FRT-moiety results in an in frame fusion of Cap-G with EGFP with a 16 aa linker in between, encoded by the NotI restriction site as well as the FRT and the Cavener sequences (Cap-G-FE; bottom). **b**, **c** Western Blot analysis of fly strains expressing the CRISPR/Cas9-generated EGFP-fusion alleles of Cap-G (**b**) and Barren (**c**). Proteins contained in extracts from two ovaries and 10 heads of individuals of the indicated genotypes were separated by SDS-PAGE, blotted, and the blots were probed with antibodies against Cap-G or Barren (top panels), against EGFP (middle panels), and against α-tubulin as loading control (bottom panels). The anti-EGFP and anti-α-tubulin probings, as well as the chemiluminescent detections were done together; identical exposure times are shown. Eye-specific EGFP expression is only detectable in extracts from heads of individuals before Flp-recombinase-mediated recombination. The signal in **b** is much weaker than in **c**, since in this case, the integrated cassette lacked the SV40 terminator region. Cap-G and Barren are much less abundant in adult heads than in ovaries, as is expected from the low abundance of the respective mRNAs in adult heads (Brown et al. 2014). Green and black arrows indicate the EGFP-fusion products and the endogenous proteins, respectively. The bands marked with asterisks are non-specific cross-reactions of the anti-Cap-G and anti-Barren antibodies in ovary extracts

In order to assess the contribution of condensin I to the fidelity of male meiotic divisions, we first investigated the localization of fluorescently tagged condensin I subunits during spermatogenesis. We have previously described functional EGFP-fused transgenes that complement the lethality associated with loss-of-function mutations of the respective condensin subunit encoding genes (Herzog et al. 2013). However, rescued animals expressing Cap-G-EGFP from classical genomic transgenes were very weak, and a genomic transgene expressing a fluorescently labeled variant of Barren was not available. To circumvent position effects of transgenes and ensure that all regulatory elements are present for proper expression, we employed the CRISPR/Cas9-system to construct strains expressing Cap-G-EGFP and Barren-EGFP in the context of the genomic loci. Despite the high efficiency of CRISPR/Cas9-induced genome changes via HDR, screening for the desired events can be quite laborious. Thus, we took advantage of an easily detectable phenotype due to fluorescent eyes (Horn et al. 2000), which has been successfully used for screening CRISPR/Cas9-mediated genome changes in a number of studies (Bosch et al. 2020; Gratz et al. 2015; Port et al. 2015). For our purpose, we combined the insertion of the screening marker with the subsequent possibility to generate translational fusions of the genes of interest with EGFP (Fig. 1; for details see “Materials and Methods”). To this end, we first introduced via CRISPR/Cas9-induced homology directed repair (HDR), a cassette directing the expression of EGFP under control of the eye-specific 3xP3-promotor immediately downstream of Barren and Cap-G. To avoid transcriptional interference of the upstream located gene with transcription initiation at the 3xP3-promotor, we inserted a fragment containing the SV40 polyadenylation (Poly(A)) signal upstream of the 3xP3-promotor. To ensure efficient translation initiation, a Cavener consensus sequence was inserted upstream the EGFP reading frame. After co-injection of the HDR templates and the gRNA-encoding plasmids into *nos-Cas9* embryos, single males were outcrossed to isolate integration events. Among the progeny, we identified individuals with green fluorescent eyes in 13% [9 out of 70 fertile crosses for *Cap-G* without the SV40 polyadenylation signal], 25% [20 out of 79 fertile crosses for *Cap-G* with the SV40 Poly(A) signal], and 28% [11 out of 39 fertile crosses for *Barren* with the SV40 Poly(A) signal] of the crosses. The presence of the SV40 Poly(A) signal greatly facilitated the identification of positive individuals due to significantly higher fluorescence intensity within the eyes. A quantification by Western Blot analysis revealed an approximately ninefold increase in EGFP-expression when compared to an insertion without the SV40 Poly(A) signal (Fig. S1). The 3xP3-promotor-cassettes are furthermore flanked by FRT-sites allowing their subsequent removal by Flp-recombinase. We have favored the Flp/FRT system over the also widely used Cre/lox system, due to potential toxic effects upon Cre recombinase expression in Drosophila (Heidmann and Lehner 2001). The FRT site immediately downstream of Cap-G or Barren was engineered to be in frame with the reading frame located upstream. As this approach removed the endogenous translational termination codon, an alternative translational termination codon was introduced right after the FRT site. The complete integrated cassettes were designated as FSV3FE for FRT-SV40 poly(A)signal-3xP3-promotor-FRT-EGFP, or F3FE for FRT-3xP3-promotor-FRT-EGFP. Embryos from established lines carrying these integrated cassettes were injected with in vitro synthesized Flp-recombinase mRNA. Upon FLP-out of the promotor-cassette, flies among the progeny will lose eye fluorescence, and concomitantly a continuous reading frame between Cap-G or Barren and EGFP is generated (Fig. 1a). The only scar left using this method is a stretch of 16 additional amino acids encoded by a NotI recognition sequence and the FRT site in between Cap-G or Barren and EGFP (Fig. 1a). Thus, the corresponding lines were designated Cap-G-FE or Barren-FE. Western blot analysis confirmed the expression of unfused EGFP in transgenic flies before FLP-out. EGFP-fused condensin subunits in addition to endogenous untagged protein were detected in heterozygous animals after FLP-out. Importantly, exclusively EGFP-fused condensin subunits were expressed in homozygous animals after FLP-out. (Fig. 1b, c). Homozygous flies harboring these constructs are healthy and fertile.

### Condensin I subunits localize to meiotic chromatin in Drosophila males

**Fig. 2:**
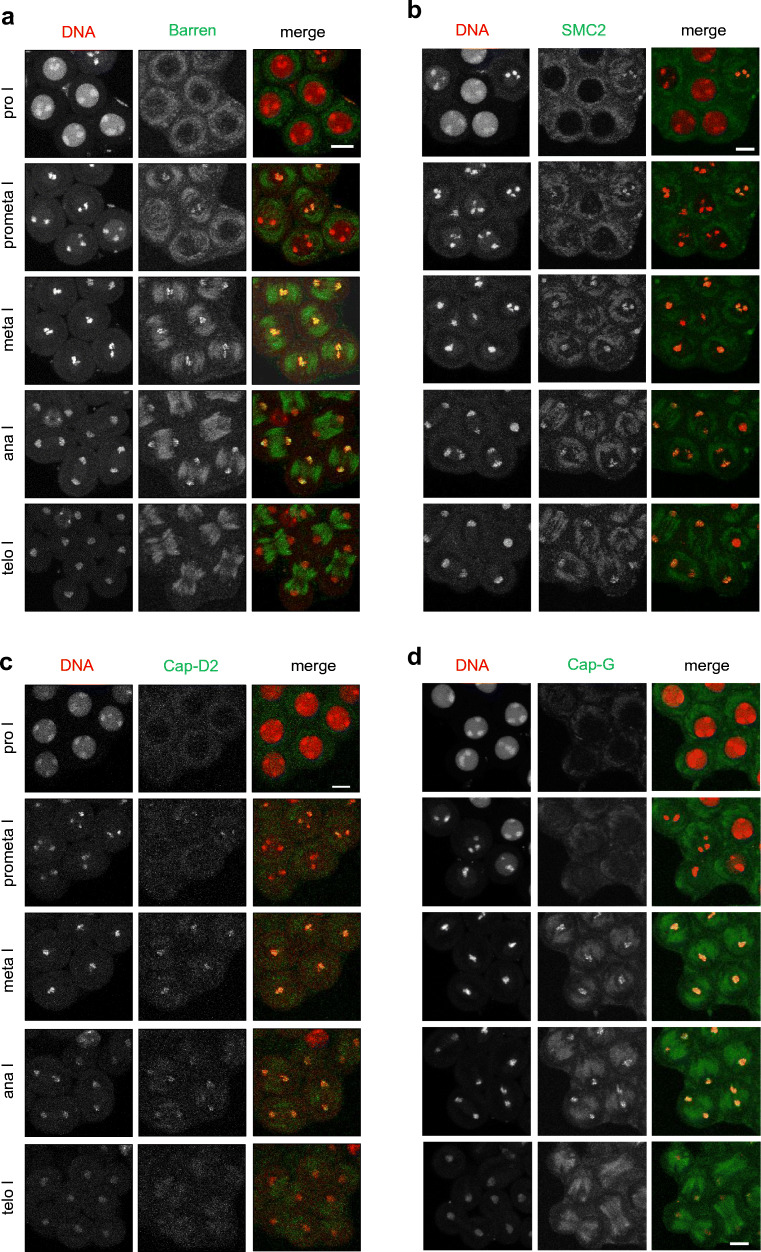
Condensin I subunits localize to spermatocyte chromatin during meiosis I. Spermatocyte cysts were prepared from pupae expressing His2Av-mRFP1 to label DNA (red in merged panels) and the EGFP-fused condensin I subunits Barren (**a**), SMC2 (**b**), Cap-D2 (**c**), or Cap-G (**d**) (green in merged panels). These subunits were expressed in an otherwise wild-type background except for SMC2_h_-EGFP, which was expressed in the presence of one mutant SMC2 allele. Cysts entering meiosis I were identified by the distinct condensed chromosome territories. Progression through prophase (pro), prometaphase (prometa), metaphase (meta), anaphase (ana), and telophase (telo) of meiosis I was monitored. Scale bars, 10 μm

The CRISPR/Cas9-generated transgenes were combined with a genomic transgene expressing red fluorescent His2Av-mRFP1 to visualize chromatin (Schuh et al. 2007). Expression, as well as localization, of the condensin subunits during male meiosis were assessed by observing developing pupal cysts in vivo. We included in these analyses genomic transgenes expressing an N-terminal EGFP-fused Cap-D2 variant or an SMC2 variant, in which EGFP was fused internally within the hinge region (SMC2_h_-EGFP). Both transgenes have been characterized previously during mitotic divisions (Herzog et al. 2013). Meiotic cysts were identified by the characteristic chromosome territories, which are formed in mid to late prophase I. At this stage, chromatin is largely devoid of a signal corresponding to the EGFP-fused condensin subunits (Fig. 2). After nuclear envelope breakdown (NEBD), which is evident by the sudden dispersal of the nucleoplasmic His2Av-mRFP1 signal that is not localized to the chromosome territories, the condensin subunits start to concentrate at the condensing meiotic chromatin. Maximal association is evident throughout metaphase I and early anaphase I, while at late anaphase I/telophase I, the fluorescence signals of the condensin subunits are largely lost from chromatin. The assessment of cytoplasmic localization was hampered by a high background fluorescence signal reminiscent of tubulin staining, which was also present in cysts from a strain that exclusively expressed His2Av-mRFP1 (Fig. S2). During progression through meiosis II, the condensin subunits localized similarly as was observed during meiosis I (Fig. S3). Thus, the localization of condensin I during the male meiotic divisions is reminiscent of the localization during mitotic cycles (Herzog et al. 2013) implying a functional importance of condensin I for Drosophila male meiosis.

### RNAi-mediated knockdown of condensin I interferes with proper meiotic chromosome segregation

**Fig. 3:**
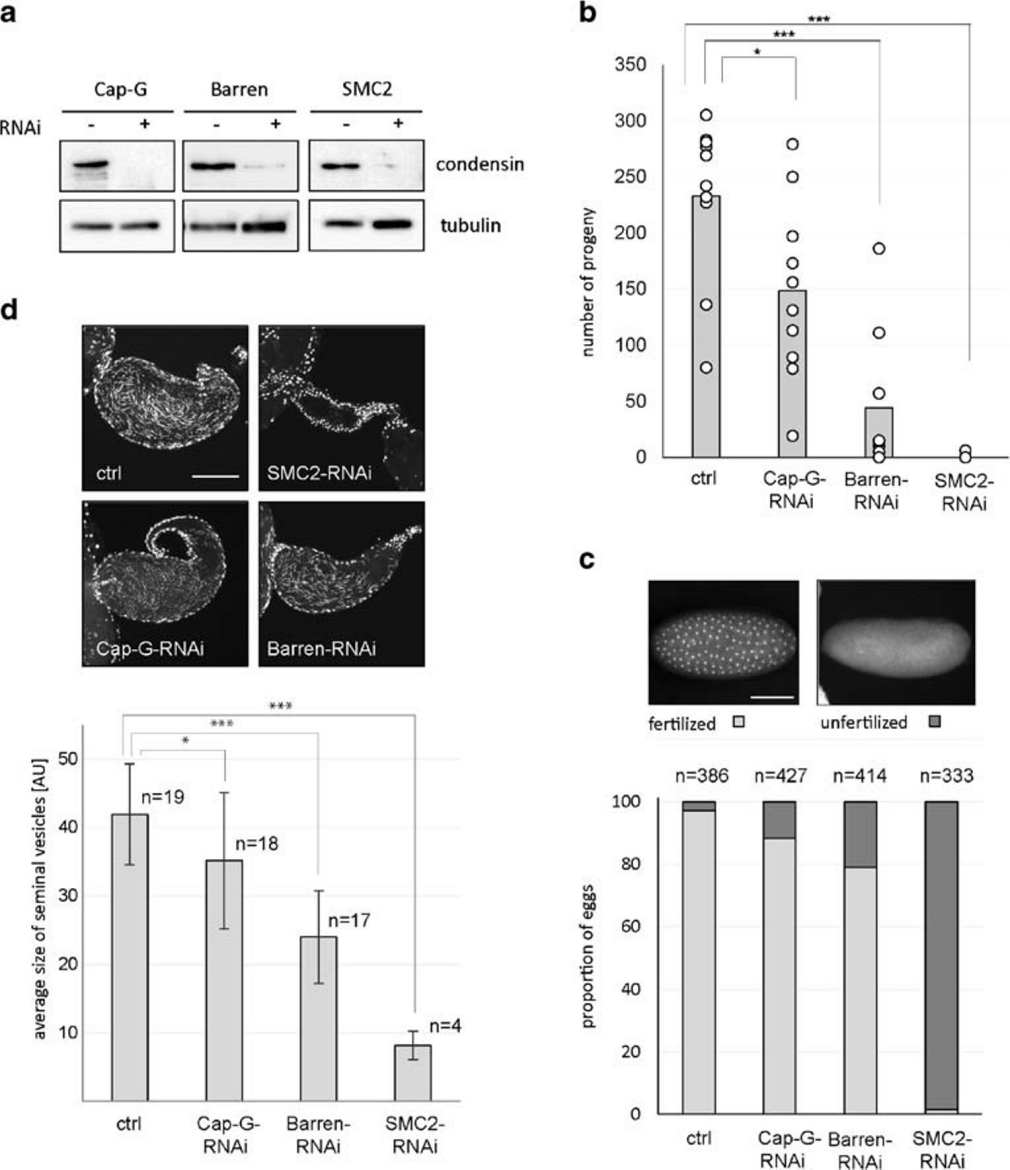
RNAi-mediated knockdown of condensin I impairs male fertility. **a** Western Blot analysis demonstrating RNAi-mediated protein depletion. Extracts were prepared from testes of individuals at the pupal stage with the genotypes *bam-GAL4-VP16* (− RNAi) or *UAS-Cap-G-RNAi/bam-GAL4-VP16* (+ RNAi) or *UAS-Barren-RNAi/+*; *bam-GAL4-VP16/+* (+ RNAi), or *UAS-SMC2-RNAi/bam-GAL4-VP16* (+ RNAi). Extracts corresponding to 10 testes equivalents were analyzed in each case. The blots were probed with the respective anti-Condensin antibodies and with anti-α-tubulin as a loading control. **b** Individual males of the genotypes *bam-GAL4-VP16* (ctrl) or *UAS-Cap-G-RNAi/bam-GAL4-VP16* (Cap-G-RNAi) or *UAS-Barren-RNAi/+*; *bam-GAL4-VP16/+* (Barren-RNAi), or *UAS-SMC2-RNAi/bam-GAL4-VP16* (SMC2-RNAi) were mated with *w*^*1*^-virigins, and the number of progeny was counted. The bars represent the mean of the progeny eclosing from ten crosses. **c** 30 males with the same genotypes as in **b** were crossed with 70 *w*^*1*^-virigins. 0–3-h-old embryos from these crosses were collected and stained for DNA. Embryos showing clear signs of development by appearance of many DNA masses were classified as fertilized and those containing just the products of female meiosis were classified as unfertilized. Scale bar, 100 μm. **d** Males with the same genotypes as in **b** and **c** were kept for 10 days in the absence of females, dissected, and the seminal vesicles were prepared and stained for DNA. The sizes of the seminal vesicles were determined and the average sizes are displayed in arbitrary units in the bar graph. Bars, mean; whiskers, S.E.M. Scale bar, 50 μm. Significances in **b** and **d** were assessed with an unpaired Student’s *t* test (*p < 0.05; ***p < 0.001)

To assess the functional relevance of condensin I for the development of productive sperm, we depleted the subunits Cap-G, Barren, and SMC2 by RNA-interference (RNAi) specifically in testes using UAS-RNAi constructs and the *bam-GAL4-VP16* driver line (Chen and McKearin 2003). This GAL4 driver line has been used previously to successfully deplete mRNAs during male meiosis in combination with UAS-RNAi transgenes (Blattner et al. 2016; Raychaudhuri et al. 2012). Western Blot analyses indeed revealed efficient reduction of condensin protein levels in pupal testes (Fig. 3a). We had also included in our experiments various UAS-RNAi transgenes targeting Cap-D2, but in none of the cases we saw significant reduction of Cap-D2 protein levels in testes (data not shown). Males expressing RNAi hairpins targeting SMC2, Cap-G, or Barren had a significantly reduced fecundity, with males expressing SMC2-RNAi being almost completely sterile (Fig. 3b). These reduced brood sizes are reflected by an increased proportion of unfertilized eggs laid by the females in these crosses (Fig. 3c) as well as reduced sizes of seminal vesicles in the males (Fig. 3d). The small seminal vesicles found in SMC2-RNAi males, which appear to be almost devoid of sperm, and the very high proportion of unfertilized eggs, perfectly correspond with the fact that these males produce almost no adult progeny. However, when Barren or Cap-G are depleted by RNAi, the number of progeny is apparently affected to a higher degree as would be suggested by the proportion of unfertilized eggs. This discrepancy could be explained by aberrant sperm capable of fertilization, but unable to support development to the adult fly. Thus, we analyzed, whether males with reduced levels of Cap-G and Barren produce aneuploid sperm by performing nondisjunction analyses for the fourth and the sex chromosomes. For the fourth chromosome nondisjunction tests, we included as control *solo*^*1*^-homozygous males. *solo* encodes a meiotic cohesion protein and its mutation results in nondisjunction at both meiosis I and II (Yan et al. 2010). The nondisjunction analyses revealed indeed that after depletion of Cap-G or Barren, a significantly elevated proportion of exceptional progeny is produced, with rates being similar to the rates obtained with the *solo* mutant males (Table 1). Our nondisjunction tests for the fourth chromosome did not allow to distinguish whether the exceptional sperm are a result from defects in meiosis I or in meiosis II. However, the diplo XY-exceptions observed in the sex chromosome nondisjunction tests can only result from nondisjunction in meiosis I (Table 2). The nullo-XY-exceptions in this analysis outnumbered by far the diplo-XY exceptions, indicating additional nondisjunction at meiosis II. However, some of these nullo-XY exceptions may also be the result of chromosome loss during any of the meiotic divisions or meiotic drive, which favors the recovery of sperm with less DNA content (Peacock et al. 1975). To specifically assess chromosome nondisjunction during meiosis II, we crossed control males and condensin-depleted males with *C(1)RM/0* females, which carry a compound X-chromosome. Patriclinious females emerging from these crosses inherit their two X-chromosomes from the father, thus reflecting meiosis II nondisjunction. Indeed, we observed a significant proportion of patriclinious female progeny after depletion of Barren or Cap-G but none in the control cross (Table 3). While in this assay, some exceptional sperm categories cannot be unambiguously identified by the phenotype of the progeny, it is important to note that all y^+^ female progeny result from fertilization of nullo-X oocytes with sperm that must originate from a nondisjunction event in meiosis II (XX, XXY, or XXYY sperm). Thus, depletion of condensin I results in nondisjunction in both meiotic divisions.

**Table 1:** Nondisjunction analysis of the fourth chromosome after RNAi-mediated depletion of condensin I subunits

Paternal genotype		Regular sperm	Exceptional sperm	Total progeny	%NDJ^1^
Chromosome 2	Chromosome 3	haplo-4	nullo-4		
+/+	+/+	1356	1	1357	0.1
*solo*^*1*^*/solo*^*1*^	+/+	335	76	411	18.5
*+/+*	*UAS-Cap-G-RNAi/bam-GAL4*	1199	313	1512	20.5
*UAS-Barren-RNAi/+*	*bam-GAL4/+*	460	77	537	14.3

Males of the indicated genotypes were crossed with *C(4) RM*, *ci*^*1*^, *ey*^*R*^ females. ^1^The percentage of nondisjunction is an underestimate as some classes of exceptional sperm could not be scored because the resulting combination is phenotypically not evident (diplo-4 or tetra-4 resulting from fertilizing a nullo-4 egg or a *C(4) RM* egg, respectively, with a diplo-4 exceptional sperm)

**Table 2:** Nondisjunction analysis of sex chromosomes after RNAi-mediated depletion of condensin I subunits

Paternal genotype			Regular sperm		Exceptional sperm		Total progeny	%NDJ^1^
Sex chromosomes	Chromosome 2	Chromosome 3	X	Y	diplo (XY)	nullo (0)		
*w*^*1*^*/Dp(1*;*Y)B*^*s*^*Yy*^*+*^	*+/+*	*+/+*	1309	1137	3	1	2450	0.2
*w*^*1*^*/Dp(1*;*Y)B*^*s*^*Yy*^*+*^	*+/+*	*UAS-Cap-G-RNAi/bam-GAL4*	474	382	12	116	984	13.0
*w*^*1*^*/Dp(1*;*Y)B*^*s*^*Yy*^*+*^	*UAS-Barren-RNAi/+*	*bam-GAL4/+*	871	778	21	74	1744	5.5

^1^The percentage of nondisjunction is an underestimate, as some classes of exceptional sperm could not be scored because the resulting combination is either lethal (triplo-X resulting from XX exceptional sperm) or phenotypically not evident (XYY resulting from YY exceptional sperm)

**Table 3:** Meiosis II-specific nondisjunction analysis of the X-chromosome after RNAi-mediated depletion of condensin I subunits. Males of the indicated genotype were crossed to *C(1) RM*, *y*^*2*^*su (w*^*a*^*)*^*1*^*w*^*a*^*/0* females. Regular sperm with one X chromosome results in y^+^ male progeny after fertilization of nullo-X oocytes. Regular sperm with one Y chromosome results in y^−^ female progeny after fertilization of C(1) RM oocytes. XX exceptional sperm results in y^+^ female progeny after fertilization of nullo-X oocytes. Similarly, XXY and XXYY sperm (as a result of nondisjunction both in meiosis I and meiosis II) results in y^+^ female progeny after fertilization of nullo-X oocytes. Exceptional nullo-XY sperm (as a result of nondisjunction in either meiosis I or meiosis II) or YY-sperm (as a result of nondisjunction in meiosis II) result in y^−^ female progeny after fertilization of C(1) RM oocytes and go undetected in this assay. Exceptional XY sperm (as a result of nondisjunction in meiosis I) or XYY sperm (as a result of nondisjunction in both meiosis I and II) result in y^+^ male progeny after fertilization of nullo-X oocytes and also go undeteted in this assay

Paternal genotype			Progeny phenotype			Total progeny	%NDJ^1^
Sex chromosomes	Chromosome 2	Chromosome 3	Male	Female			
			y^+^	y^+^	y^−^		
*w*^*1*^*/Y*	*+/+*	*bam-GAL4/bam-GAL4*	353	0	405	758	0
*w*^*1*^*/Y*	*+/+*	*UAS-Cap-G-RNAi/bam-GAL4*	184	53	457	694	7.6
*w*^*1*^*/Y*	*UAS-Barren-RNAi/+*	*bam-GAL4/+*	230	203	204	637	32.0

^1^ The percentage of meiosis II nondisjunction is an underestimate, as some classes of exceptional sperm could not be scored because the resulting combination is phenotypically not evident (y^−^ - females with the karyotype XXYY resulting from YY exceptional sperm fertilizing C(1) RM oocytes and y^+^ - males with the karyotype XYY resulting from XYY exceptional sperm fertilizing nullo-X oocytes)

**Fig. 4:**
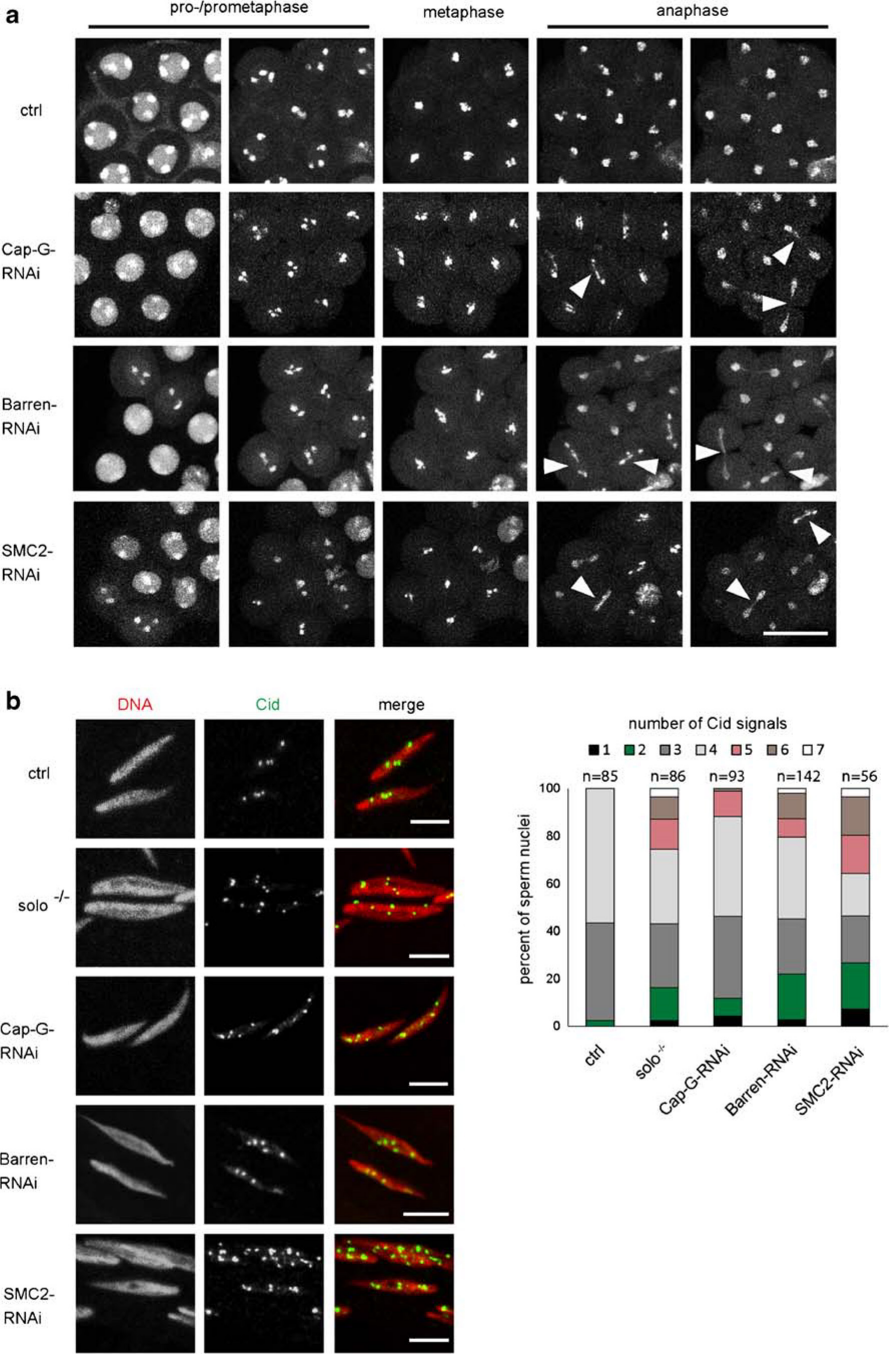
Condensin I depletion results in defective male meiosis. **a** Spermatocyte cysts were prepared from pupae with the genotype *His2Av-mRFP1*, *bam-GAL4-VP16/+* (ctrl) or *UAS-Cap-G-RNAi/His2Av-mRFP1*, *bam-GAL4-VP16* (Cap-G-RNAi) or *UAS-Barren-RNAi/+*; *His2Av-mRFP1*, *bam-GAL4-VP16/+* (Barren-RNAi), or *UAS-SMC2-RNAi/His2Av-mRFP1*, *bam-GAL4-VP16* (SMC2-RNAi). Cysts entering meiosis I were identified by the distinct condensed chromosome territories as revealed by chromatin-associated His2Av-mRFP1. Progression through prophase/prometaphase, metaphase, and anaphase was monitored by in vivo microscopy. Arrowheads indicate anaphase bridges. Scale bar, 20 μm. **b** Squashed adult testes were fixed and stained for DNA (red in merged images) and for CENP-A/Cid (green in merged images) to assess aneuploidy in sperm nuclei. Testes were prepared from individuals with the genotypes *bam-GAL4-VP16* (ctrl) or *solo*^*Z2–0198*^*/solo*^*Z2–0198*^ (solo^−/−^) or *UAS-Cap-G-RNAi/bam-GAL4-VP16* (Cap-G-RNAi) or *UAS-Barren-RNAi/+*; *bam-GAL4-VP16/+* (Barren-RNAi), or *UAS-SMC2-RNAi/bam-GAL4-VP16* (SMC2-RNAi). The number of CENP-A/Cid signals in each sperm nucleus was determined

**Fig. 5:**
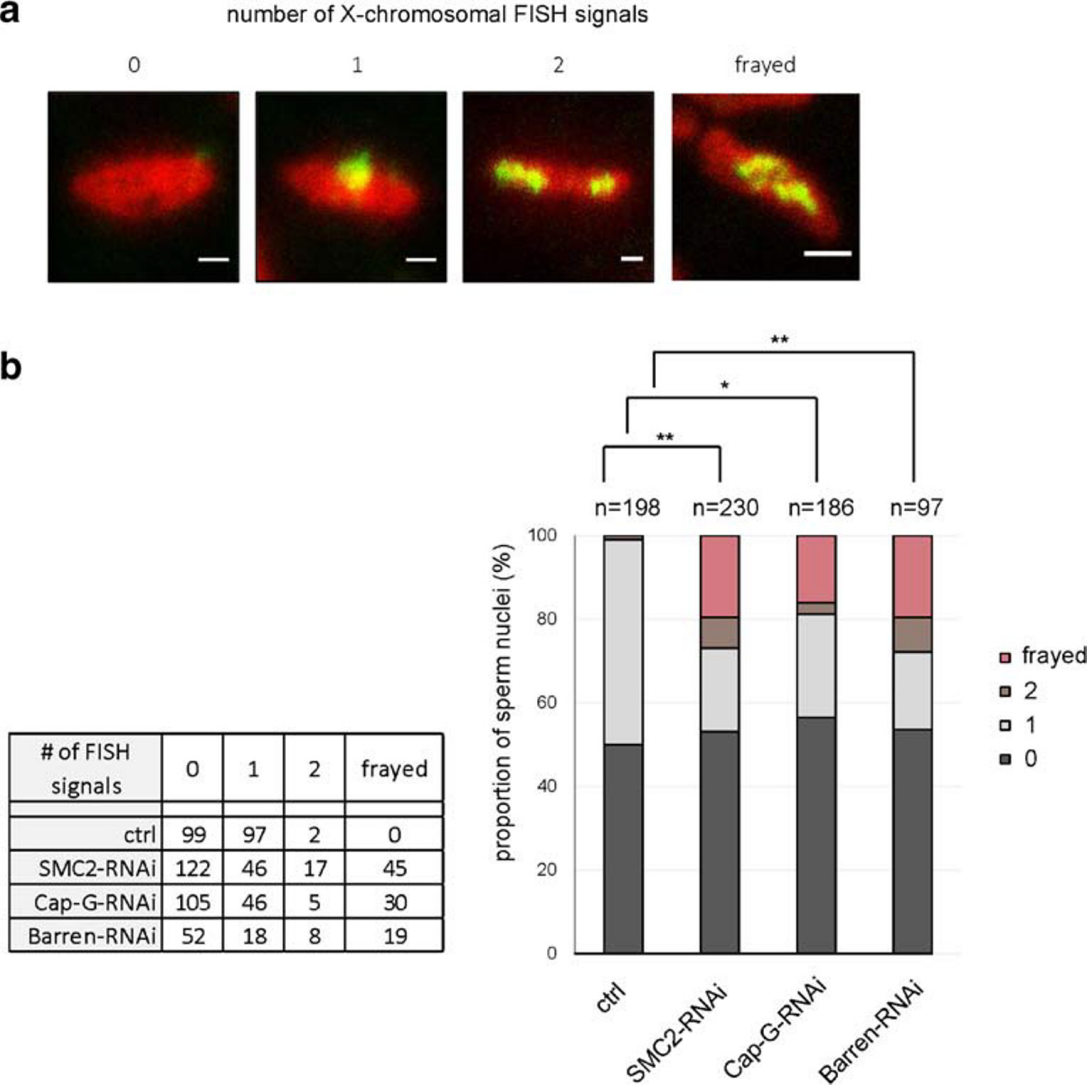
Condensin I depletion results in spermatids with aberrant number of X-chromosomal FISH signals. Adult testes were prepared, squashed, and hybridized with a X-chromosomal FISH probe. **a** Images of individual spermatid nuclei representative of the four classes that were defined to describe the observed FISH signal distributions. Red, DNA stained with Hoechst 33258; green, FISH signal. Scale bars, 2 μm. **b** Numbers and proportion of spermatids assigned to the individual classes, prepared from males with the following genotypes: *bam-GAL4-VP16* (ctrl), or *UAS-SMC2-RNAi/bam-GAL4-VP16* (SMC2-RNAi) or *UAS-Cap-G-RNAi/bam-GAL4-VP16* (Cap-G-RNAi) or *UAS-Barren-RNAi/+*; *bam-GAL4-VP16/+* (Barren-RNAi). The proportion of spermatids with two signals after condensin I depletion was significantly higher when compared with the control (*p < 0.05; **p < 0,005; chi-square goodness of fit test)

To directly visualize chromatin organization and chromosome segregation errors during the meiotic divisions, we performed live cell microscopy on cysts derived from males expressing the condensin RNAi constructs as well as red fluorescent His2Av-mRFP1 to visualize chromatin (Fig. 4a). We noticed in all depletion scenarios that meiosis I prophase nuclei could be identified by the presence of the chromosome territories reflecting the conjoined second and third chromosomes, as well as the gonosomes. These territories appeared less pronounced in the case of Barren-RNAi reminiscent of the phenotype in mutants of the condensin II subunit encoding genes *Cap-H2* and *Cap-D3*, where chromosome territories are virtually absent (Hartl et al. 2008b). Since in our in vivo experiments, DNA is indirectly labeled via association with His2Av-mRFP1, we also analyzed prophase I nuclei in fixed samples stained with a DNA dye to scrutinize chromosome territory formation (Fig. S4a). In all cases, we could unambiguously identify three major separate DNA masses representing the territories typical for prophase of male meiosis I. Thus, formation of chromosome territories does not appear to be disturbed significantly by RNAi-mediated knockdown of Cap-G, Barren, or SMC2. However, after depletion of any of the three components, chromatin bridges are evident in cysts progressing through anaphase of meiosis I (Fig. 4a). While we have not observed these bridges in control cells (92 cells; 6 cysts), they were abundant, when condensin subunits were depleted: Cap-G RNAi: 50% cells with bridges (38 out of 76 cells; 6 cysts); Barren RNAi: 48% cells with bridges (38 out of 79 cells; 5 cysts); SMC2 RNAi: 86% cells with bridges (54 out of 63 cells; 5 cysts). Such chromatin bridges were also observed in immunofluorescence analyses of fixed adult testes (Fig. S4b). Furthermore, chromatin bridges were also abundant during anaphase of meiosis II upon depletion of condensin I, as analyzed by in vivo imaging (Fig. S5). In control cysts progressing through meiosis II, we observed anaphase bridges in 6% of the cells (7 out of 118 cells; 4 cysts), but many more upon depletion of condensin I: Cap-G RNAi: 65% cells with bridges (65 out of 100 cells; 5 cysts); Barren RNAi: 74% cells with bridges (94 out of 127 cells; 5 cysts); SMC2 RNAi: 88% cells with bridges (110 out of 125 cells; 6 cysts). If these chromatin bridges during both meiotic divisions are indicative of missegregation, one would expect sperm to be formed with an aberrant number of chromosomes. To analyze chromosome number, we performed squash preparations of adult testes and stained for DNA and the centromere marker Cid (Henikoff et al. 2000). In the wildtype control, 56% of the sperm contained four separate Cid signals, and the remainder three or two spots (Fig. 4b). This is consistent with the expectation, since in the cases with less than four spots, the Cid signals of different chromosomes most probably were superimposed. Importantly, we never observed more than four Cid signals in sperm nuclei from control males. However, after condensin depletion, between 12 and 36% of the sperm contained more than four signals, which is in the same order of magnitude when compared to *solo*-mutant males (26%) (Fig. 4b). To analyze the segregation behavior of an individual chromosome, we performed fluorescence in situ hybridization (FISH) specific for the X-chromosome on squashed preparations of adult testes. In the control (no UAS-transgene), about 50% of the sperm nuclei were devoid of an X-chromosome specific signal, and the great majority of the residual sperm nuclei contained a single, clearly defined spot, as expected. However, upon depletion of Cap-G, Barren, and SMC2, we observed in 3% (5 out of 186), 8% (8 out of 97), and 7% (17 out of 230), respectively, of the sperm nuclei two separate FISH-signals indicative of two X-chromosomes (Fig. 5). In the control, only 1% (2 out of 198) of the sperm nuclei exhibited two X-chromosomal FISH signals. The significantly increased occurrence of two X-chromosomal signals is a clear indication of increased nondisjunction in meiosis II upon condensin I knockdown and is consistent with the genetic test for meiosis II nondisjunction (Table 3). Furthermore, in 16–20% of the condensin-depleted sperm nuclei, the X-chromosome-specific FISH signal was spread out and had a frayed appearance. In these cases, it is difficult to judge whether there are two or more (overlapping) signals, or whether the signal is due to defective compaction of a single X-chromosome. However, given the high levels of nondisjunction in meiosis II, a substantial proportion of the “frayed” class probably represents nuclei with two X-chromosomes. Significantly, we did not observe such a phenotype in the sperm nuclei of the control (Fig. 5). Taken together, our RNAi-depletion studies suggest that condensin I is required for faithful chromosome segregation during both meiotic divisions in Drosophila males.

### Induced proteasomal degradation of Barren results in reduced male fertility

The meiotic phenotypes observed after condensin depletion via RNAi could theoretically be due to off-target effects. We tried to rescue these phenotypes by expressing RNAi-resistant transgenes in the male germline, but several attempts failed to construct the required strains containing multiple transgene insertions. However, we were able to effectively rescue the lethality associated with expression of the condensin RNAi-constructs in mitotically dividing tissue using the *ey-GAL4* driver line, when simultaneously expressing RNAi-resistant transgenes, but not when expressing RNAi-sensitive transgenes containing the wild-type sequence (Fig. S6). Although this experiment, as well as the very similar phenotypes obtained after downregulating different condensin I-subunits by RNAi, significantly reduce the likelihood that the meiotic phenotypes are caused by off-target effects, such effects cannot be completely excluded. Thus, we set out to interfere with condensin function during male meiosis using a different method. We took advantage of the specific proteasomal degradation of GFP-fused proteins with the DeGradFP-System (Caussinus and Affolter 2016). In this setup, GFP-fused target proteins are recognized by a GFP-specific nanobody (vhhGFP4), which itself is fused to the N-terminally localized F-Box region of Slmb (NSlmb), thus recruiting the target protein to the SCF E3-ubiquitin ligase and triggering its degradation in the 26S proteasome. It has been demonstrated previously that this system can be used to significantly reduce levels of EGFP-fused target proteins not only in mitotically proliferating cells, but also in the female and male germlines (Raychaudhuri et al. 2012; Sun et al. 2019; Urban et al. 2014). Indeed, the NSlmb-vhhGFP4-dependent depletion of Barren-EGFP in testes of Barren-FE expressing males was obvious upon Western blot analysis (Fig. S7b). Barren-FE homozygous males expressing *NSlmb-vhhGFP4* under control of *bam-GAL4* produced on average 46 progeny in the fertility test. In contrast, a group of control males lacking the *UAS-NSlmb-vhhGFP4* transgene produced on average 85 progeny (Fig. S7a). A biological replicate of this experiment yielded very similar results (data not shown). The results imply that the reduction of Barren has a negative impact on male fertility. To determine whether the reduced fertility coincides with defects in meiosis, we analyzed meiosis I anaphase figures in fixed adult testes by immunofluorescence. We indeed observed a higher proportion of anaphase bridges in adult testes in the samples when Barren-FE was depleted using the DegradFP-system (five cells with bridges in 13 anaphase figures), when compared to samples from the control group lacking *UAS-NSlmb-vhhGFP4* (one cell with a bridge in 18 anaphase figures) (Fig. S7c). This difference is significant (*p* < 0.01; chi-square goodness of fit test). Taken together, our data thus strongly support an essential role of condensin I for the accuracy of male meiotic divisions.

## Discussion

Our aim of the current study was to assess the contribution of condensin I to the faithfulness of male meiosis in Drosophila. For analysis of the localization behavior of Barren and Cap-G during the meiotic divisions, we generated genomic EGFP knock-in strains using the CRISPR/Cas9-system. Despite the high efficiency of CRISPR/Cas9-mediated genome manipulation, screening of the desired events can be quite laborious. We have taken advantage of the easy detection of integration events by scoring eye fluorescence as a marker, which has been used as a screening marker in several other CRISPR/Cas-based genomic tagging strategies (Bosch et al. 2020; Gratz et al. 2015; Port et al. 2015). The percentage of crosses with positive (EGFP+) founder males among the progeny (between 13 and 28%) was lower than in a comparable setup, where the HDR-template and a gRNA-encoding plasmid were also co-injected in *nos-Cas9* expressing individuals (between 46 and 88% crosses with founder males) (Port et al. 2015). This difference could be due to varying efficiencies of the targeting potential of the different gRNAs used. However, the numbers are still high enough to allow for screening for integration events with modest effort. Importantly, subsequent Flp-recombinase-mediated excision of the eye-specific promotor-cassette results in the generation of an in-frame fusion of the gene of interest with EGFP. Thus, loss of eye fluorescence indicates the generation of the desired fusion construct and the screening marker is converted to the fusion tag. Flp- or Cre-recombinase-mediated removal of marker genes is a common strategy (Gratz et al. 2015; Kunzelmann et al. 2016), which in our case is not necessary since the marker gene serves a different purpose after FLP-out of the promotor-cassette. We could confirm the synthesis of Cap-G-EGFP and Barren-EGFP by Western Blot analysis and our in vivo imaging, arguing against ectopic integration of the donor plasmids elsewhere in the genome. We have delivered Flp-recombinase by injecting in vitro synthesized mRNA into embryos containing the FRT cassettes. Alternatively, one could employ strains expressing FLPase under control of an inducible promotor (e.g., hs-FLP) and screen among the F2-progeny for individuals having lost eye fluorescence. In our hands, we obtained higher efficiencies of FLP-out after mRNA injection in two experiments, so that we used mRNA injection throughout our study. We also observed significantly higher 3xP3-driven EGFP expression when we inserted the SV40 terminator region upstream of 3xP3 promoter. While we have directly compared the effects ± SV40 terminator only in the context of *Cap-G*, we suspect a general advantage of blocking read-through transcription of the upstream-located gene of interest. Our approach complements genome-wide protein traps, which are designed to internally tag proteins of interest by inserting the reading frames of fluorescent proteins as artificial exons, flanked by splice acceptor and splice donor sites, into introns (Buszczak et al. 2007; Morin et al. 2001; Quinones-Coello et al. 2007). The protein traps leave basically no scar except for the desired fusion with the fluorescent protein reading frame upon splicing. In our approach, after Flp-recombinase-mediated excision of the promoter cassette, the remaining FRT site and a restriction site encode additional 16 amino acids. We cannot rule out that this additional amino acid stretch may negatively influence the function of the desired fusion protein of interest in some cases. However, if the protein retains its functionality with an immediate fused C-terminal EGFP tag, those additional 16 amino acids between EGFP and the reading frame of the gene of interest most probably will not be deleterious. Our C-terminal tagging approach certainly can be modified using any fluorescent protein as fusion partner. We would like to point out that our approach is easily applicable for C-terminal fusions only, as N-terminal or internal fusions would require synthesis of fusion proteins under control of the 3xP3 promoter and after FLP-out the remaining FRT site may interfere with transcription and/or translation initiation. Furthermore, while after insertion of the cassette downstream of the gene of interest, this gene is still expressed under control of its 5′- and internal regulatory elements, N-terminal or internal tagging could interfere with expression and would result in the ectopic and immediate synthesis of tagged full-length protein or protein fragments, respectively. Whether C-terminal tagging influences the function of the protein of interest, needs to be assessed from case to case. However, if C-terminal tagging with a fluorescent protein is deemed appropriate, our approach is a convenient tool complementing existing methods for fluorescent tagging of target proteins using the CRISPR/Cas9-system (Bosch et al. 2020; Li-Kroeger et al. 2018).

The localization of all condensin I-specific subunits to chromatin in both meiotic divisions implies a functional role. Indeed, we observed after RNAi-mediated knockdown of the condensin I specific subunits Cap-G and Barren anaphase bridges, a phenotype which is very similar to what is observed in cells progressing through mitosis with a reduced amount of condensin I complex components (Bhat et al. 1996; Dej et al. 2004; Jäger et al. 2005; Oliveira et al. 2005; Savvidou et al. 2005; Steffensen et al. 2001). These anaphase bridges were observed in both meiosis I and meiosis II. Anaphase bridges in meiosis I are consistent with the occurrence of XY-exceptional sperm indicating nondisjunction events in meiosis I. Anaphase bridges in meiosis II are consistent with the occurrence of two X-chromosomal signals in our FISH assays indicating nondisjunction events in meiosis II, which is also supported by our meiosis II-specific nondisjunction results. Condensin complexes are required in mitotic cells, in cooperation with topoisomerase II, to resolve sister chromatids which are catenated due to DNA replication in the preceding S-phase (Piskadlo and Oliveira 2017). Homologous chromosomes paired in meiosis I do not originate from DNA replication, yet their DNA appears entangled in anaphase I in the absence of condensin. In budding yeast, these anaphase bridges in meiosis I were shown to depend on meiotic recombination (Yu and Koshland 2003). However, condensin depletion-dependent anaphase bridges during meiosis in *Tetrahymena thermophila* are independent of meiotic recombination (Howard-Till and Loidl 2018), and Drosophila males show such bridges in the complete absence of recombination after depletion of condensin I (this study) and also condensin II (Hartl et al. 2008b). The bridges identified in the latter study could be attributed to the association of both homologous and heterologous chromosomes (Hartl et al. 2008b). Most probably, the lack of proper chromatin organization under conditions, when condensin is downregulated, leads to a spatial overlap of both homologous and heterologous chromatin regions, upon which topoisomerase II can act and introduce catenations (Piskadlo and Oliveira 2017). Those catenations are not timely resolved and lead to anaphase bridging in the subsequent anaphase. Alternatively, bridging could also be due to persistent chromatin associations mediated by protein complexes such as the autosome-specific Teflon-mediated homolog conjunction complex (Arya et al. 2006; Hartl et al. 2008b). Regardless of the mechanistic basis, as a consequence of the chromatin bridges, many sperm nuclei contained an aneuploid set of chromosomes as shown by our nondisjunction analyses, the FISH-experiments, and also the number of centromeric CENP-A/Cid-signals.

The phenotypes we observed after RNAi-mediated knockdown of SMC2 were more severe than after knockdown of Cap-G or Barren. This could be due to variable knockdown efficiencies, but our Western blot analyses suggest very similar levels of protein depletion (Fig. 3a). As SMC2 is a component of both condensin I and condensin II, depletion of SMC2 would affect both condensin complexes, while depletion of Cap-G and Barren affects only condensin I. Since *Cap-D3* and *Cap-H2* mutants have been found to result in complete male sterility (Hartl et al. 2008b; Savvidou et al. 2005), the sterility we observed after SMC2 knockdown can easily be explained by loss of condensin II function. *Cap-D3* and *Cap-H2* mutant males are also characterized by highly irregular chromosome territory formation during prophase I (Hartl et al. 2008b). Thus, it is surprising that chromosome territories are obvious and look normal after knockdown of SMC2. One possible explanation for this discrepancy is that Cap-D3 and Cap-H2 may function in the process of territory formation outside the context of a bona fide condensin II complex, i.e., without SMC2. While in some cell types the proposed anti-pairing activity of condensin II subunits does depend on the SMC subunits (Hartl et al. 2008a), it remains a possibility that Drosophila Cap-H2 and Cap-D3 function in territory formation in a different context. Alternatively, it is possible that residual SMC2 protein levels remaining after RNAi-mediated knockdown suffice to convey early condensin II function during prophase I (i.e., presence of intact territories) but are insufficient for proper condensin I and II function during later stages. In our in vivo experiments, the chromosome territories appear less obvious after knockdown of Barren, and a relatively high His2Av-mRFP1 signal is present in the nucleoplasm (Fig. 4a) suggesting a function for Barren in chromosome territory formation and/or maintenance. However, after nuclear envelope breakdown, most of the nucleoplasmic staining dissipated, and clear and distinct territories are visible (see the first image in the panel for Barren-RNAi in Fig. 4a). Since clear territories can be observed in all knockdown scenarios in fixed specimen (Fig. S4a), the diffuse nucleoplasmic His2Av-mRFP1 signal after Barren knockdown in vivo may not properly reflect the organization of chromosomes within the territories. This pool of His2Av-mRFP1 may be associated with the unwound Y-chromosome loops (Bonaccorsi et al. 1988) or might not be associated with chromatin. It remains to be elucidated, why such an imbalance of His2Av-mRFP1 distribution specifically occurs after knockdown of Barren.

Obviously, condensin I cannot suppress the effects of a lack of condensin II, as *Cap-D3* and *Cap-H2* null mutant males are completely sterile despite the presence of functional condensin I. This lack of complementation may be caused by putatively post-meiotic functions of condensin II, which are unrelated to shaping meiotic chromatin (Hartl et al. 2008b). It is important to note that Drosophila condensin II, like the condensin II-complexes in a number of other insect clades, lacks the subunit Cap-G2 (Herzog et al. 2013; King et al. 2019). Since Cap-G knockdown does not result in more severe phenotypes than knockdown of Barren, it is unlikely that Cap-G takes over the function of Cap-G2 and is part of both condensin complexes, consistent with our previous report (Herzog et al. 2013). The division of labor of the two condensin complexes during male meiosis remains to be elucidated. Appropriate chromosome organization achieved in the extended interphase preceding male meiosis by the action of condensin II may be a prerequisite for proper action of condensin I during the meiotic divisions. Since we cannot exclude in our depletion experiments that residual amounts of condensin I allow the formation of some functional sperm, an absolute requirement of condensin I for male meiosis remains a possibility.

Taken together, our studies clearly demonstrate the vital importance of condensin I for proper segregation of the genetic material during the meiotic divisions in Drosophila males. Future studies will help to define the mechanistic interplay of condensin I and condensin II, assuring proper chromatin organization and chromosome segregation during the meiotic divisions in Drosophila males.

## Electronic supplementary material

Fig. S1 The integration of the SV40 transcriptional terminator within the FRT-3xP3-FRT-EGFP (F3FE) reporter cassette enhances EGFP expression approximately by nine-fold. **a)** Eye fluorescence of animals with the SV40-terminator containing FSV3FE cassette integrated downstream of the Cap-G gene (+ SV40), or with the F3FE cassette lacking the SV40 terminator region integrated downstream of Cap-G (-SV40) or of *w*^*1*^ control flies (ctrl) was monitored. **b)** Extracts were prepared from adult heads of individuals with the genotype w*; Cap-G-FSV3FE/CyO, P [ry+, ftz-lacZ] (+SV40) or from individuals with the genotype w*; Cap-G-F3FE/CyO, P [ry+, ftz-lacZ] (-SV40). In the undiluted lanes (undil.) extract corresponding to 10 head equivalents was loaded. Proteins were separated by PAGE, blotted and the blot was probed with antibodies against EGFP (top panel), and against α-tubulin as loading control (bottom panel) (PNG 273 kb).

High resolution image (TIF 2162 kb).

Fig. S2 Background signal in meiotic spermatocyte cysts when irradiated with the 488 nm laser line. Cysts were prepared from male pupae with the genotype *Sco/CyO, P [ry+, ftz-lacZ]*; *His2Av-mRFP1,* thus lacking any GFP-fused proteins*,* and observed while progressing through meiosis I. The conditions were identical to those used for generating the images shown in Fig. 2. Note the distinct cytoplasmic staining reminiscent of tubulin signals in later stages, and also the absence of any signals colocalizing with meiotic chromatin. Scale bar, 10 μm (PNG 547 kb).

High resolution image (TIF 2439 kb).

Fig. S3 Condensin I subunits localize to spermatocyte chromatin during meiosis II. Spermatocyte cysts were prepared from pupae expressing His2Av-mRFP1 to label DNA (red in merged panels) and the EGFP-fused condensin I subunits Barren (**a**) or SMC2 (**b**) or Cap-D2 (**c**) or Cap-G (**d**) (green in merged panels). These subunits were expressed in an otherwise wild-type background except for SMC2_h_-EGFP, which was expressed in the presence of one mutant SMC2 allele. Cysts completing meiosis I were identified. Progression through prophase (pro), prometaphase (prometa), metaphase (meta), anaphase (ana) and telophase (telo) of meiosis II was then monitored. Scale bar, 10 μm (PNG 5166 kb).

High resolution image (TIF 16868 kb).

Fig. S4 Phenotypic consequences on meiosis I after depletion of condensin I subunits. **a)** Chromosome territories in prophase I appear normal upon condensin I depletion. **b)** Anaphase bridges in meiosis I are frequently present after depletion of condensin I subunits. Arrowheads highlight anaphase bridges. Testes from adult males of the genotypes *w*^*1*^ (ctrl), *UAS-Cap-G-RNAi/bam-GAL4-VP16* (Cap-G-RNAi), *UAS-Barren-RNAi/+; bam-GAL4-VP16/+* (Barren-RNAi) or *UAS-SMC2-RNAi/bam-GAL4-VP16* (SMC2-RNAi) were prepared, fixed, and stained with anti-α-tubulin antibodies or Hoechst 33258 to label DNA (red in the merged panels). Scale bars, a): 25 μm; b): 10 μm (PNG 2355 kb).

High resolution image (TIF 7932 kb).

Fig. S5 Anaphase bridges in meiosis II are frequently present after depletion of condensin I subunits. Spermatocyte cysts were prepared from pupae with the genotype *His2Av-mRFP1, bam-GAL4-VP16* (ctrl) or *UAS-Cap-G-RNAi/His2Av-mRFP1, bam-GAL4-VP16* (Cap-G-RNAi) or *UAS-Barren-RNAi/+; His2Av-mRFP1, bam-GAL4-VP16/+* (Barren-RNAi), or *UAS-SMC2-RNAi/His2Av-mRFP1, bam-GAL4-VP16* (SMC2-RNAi). Cysts entering meiosis II were identified by the number and the size of the nuclei within the cysts, as revealed by chromatin-associated His2Av-mRFP1. Progression through meiosis II was monitored by in vivo microscopy. Three examples for anaphase figures of each genotype are shown. Arrowheads indicate examples of anaphase bridges. Scale bar, 25 μm. (PNG 3.41 mb).

High resolution image (TIF 6.56 mb).

Fig. S6 Specificity of phenotypes caused in mitotic proliferating tissue by RNAi-induced downregulation of condensin I function. **a)** Schematic representation of the final cross to assess suppression of RNAi-induced phenotypes. Condensin-EGFP is an abbreviation for either a wild-type (RNAi-sensitive) or RNAi-resistant transgene variant of the three analyzed condensin genes. The transgenes are expressed under control of the genomic flanking sequences, and they are all inserted at the same genomic position on the third chromosome (68E) via the φC31 integrase system. UAS-Condensin-siRNA represents transgenes expressing double-stranded RNAs under UAS control targeting SMC2, Cap-G or Barren. These transgenes are located on either chromosome II (Barren) or chromosome III (SMC2, Cap-G). **b)** Schematic representation of the progeny classes expected from the cross shown in a). The various chromosome combinations result in expression of neither siRNA nor condensin-EGFP (class I), expression of only condensin-EGFP (class II), expression of only siRNA (class III) or expression of both siRNA and condensin-EGFP (class IV, green shading). **c)** Assignments of the individuals resulting from the crosses to the various progeny classes. The expression of the siRNAs under control of the *ey-GAL4* driver result in complete (SMC2 and Cap-G) or almost complete (Barren) lethality, when only endogenous condensin is expressed (class III). Transgenic expression of only the RNAi-resistant variants significantly rescues this lethality (class IV). The few individuals which eclosed in the absence of an RNAi-resistant transgene in the case of Barren, point to an incomplete destruction of the corresponding mRNA. However, these individuals were all characterized by severe malformations of the eyes, which was observed in only the minority of the cases, when the RNAi-resistant transgene was expressed. (DOCX 14 kb).

Fig. S7 Condensin I depletion via induced proteasomal degradation results in reduced male fertility and chromatin bridges during meiosis. **a)** Individual males of the genotypes *Barren-FE; UASP-NSlmb-vhh4-GFP/TM3, Sb* (-NSlmb) or *Barren-FE; UASP-NSlmb-vhh4-GFP/bam-GAL4-VP16* (+NSlmb), were mated with *w*^*1*^-virigins, and the number of progeny was counted. **b)** Western Blot analysis of fly strains expressing Barren-FE exclusively (homozygous), or in the presence of one wild-type allele (heterozygous) in the presence (+) or absence (−) of NSlmb-vhh4-GFP. The green and the black arrow indicate the EGFP-fusion products and the endogenous proteins, respectively. **c**) Testes from adult males of the genotypes *Barren-FE; UASP-NSlmb-vhh4-GFP/TM3, Sb* (-NSlmb) or *Barren-FE; UASP-NSlmb-vhh4-GFP/bam-GAL4-VP16* (+NSlmb) were prepared, fixed, and stained with anti-α-tubulin antibodies or Hoechst 33258 to label DNA (red in the merged panels). Anaphase bridges were more frequently observed in the presence of NSlmb-vhh4-GFP (5 out of 13 anaphase cells) when compared to the absence of NSlmb-vhh4-GFP (1 out of 18 cells). Examples for anaphase figures are shown in the enlarged panels on the right. Scale bars, 10 μm (PNG 718 kb).

High resolution image (TIF 3440 kb).
