# Supplementary figures and images for: Condensin I is required for faithful meiosis in Drosophila males
Source: Chromosoma. 2020 Apr 8;129(2):141–60. doi: 10.1007/s00412-020-00733-w (PMC7260282; doi:10.1007/s00412-020-00733-w)

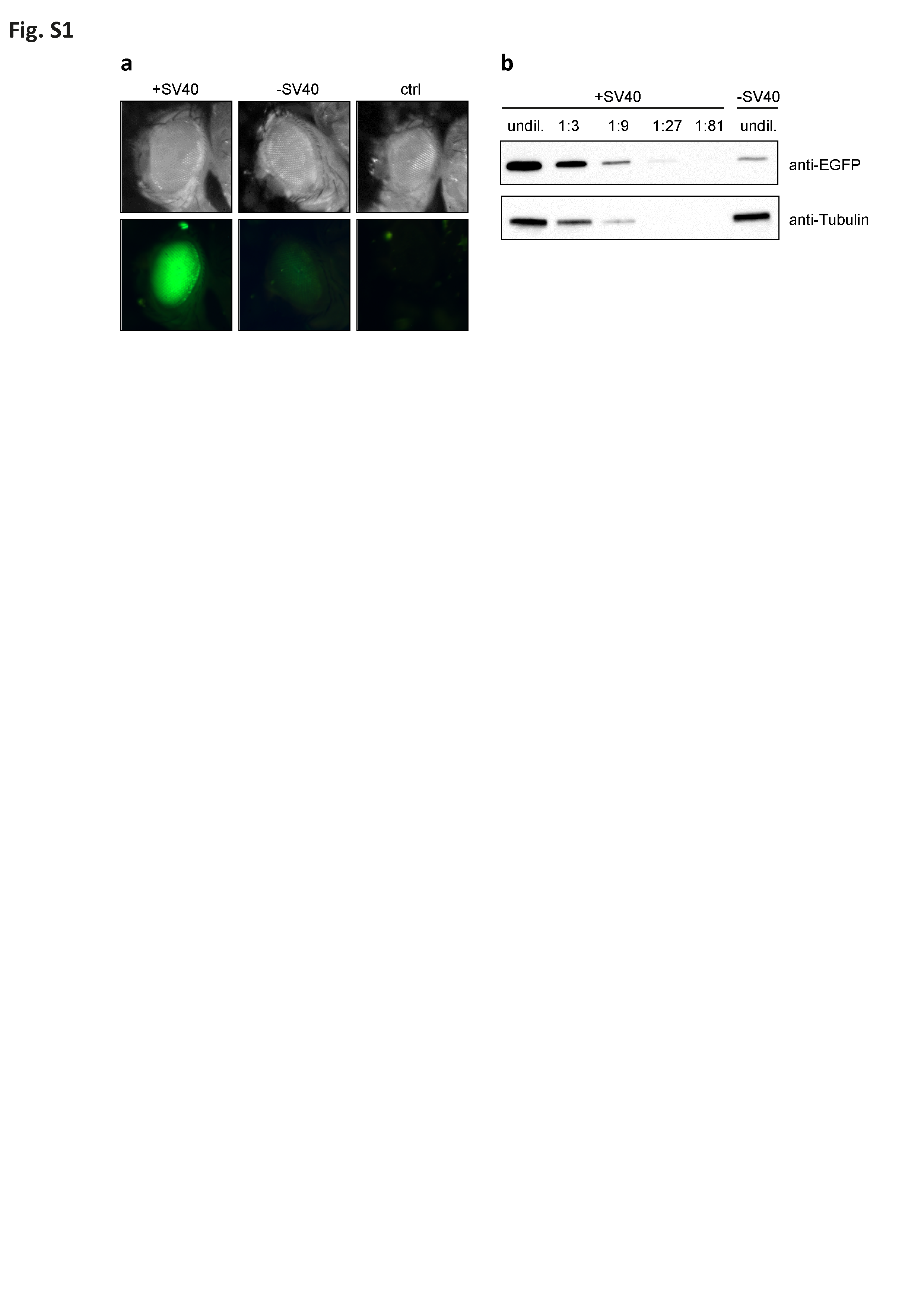

Supplement: Supplementary file 1 — The integration of the SV40 transcriptional terminator within the FRT-3xP3-FRT-EGFP (F3FE) reporter cassette enhances EGFP expression approximately by nine-fold. a) Eye fluorescence of animals with the SV40-terminator containing FSV3FE cassette integrated downstream of the Cap-G gene (+ SV40), or with the F3FE cassette lacking the SV40 terminator region integrated downstream of Cap-G (-SV40) or of w1 control flies (ctrl) was monitored. b) Extracts were prepared from adult heads of individuals with the genotype w*; Cap-G-FSV3FE/CyO, P [ry+, ftz-lacZ] (+SV40) or from individuals with the genotype w*; Cap-G-F3FE/CyO, P [ry+, ftz-lacZ] (-SV40). In the undiluted lanes (undil.) extract corresponding to 10 head equivalents was loaded. Proteins were separated by PAGE, blotted and the blot was probed with antibodies against EGFP (top panel), and against α-tubulin as loading control (bottom panel) (PNG 273 kb). [file 412_2020_733_Fig6_ESM.png]

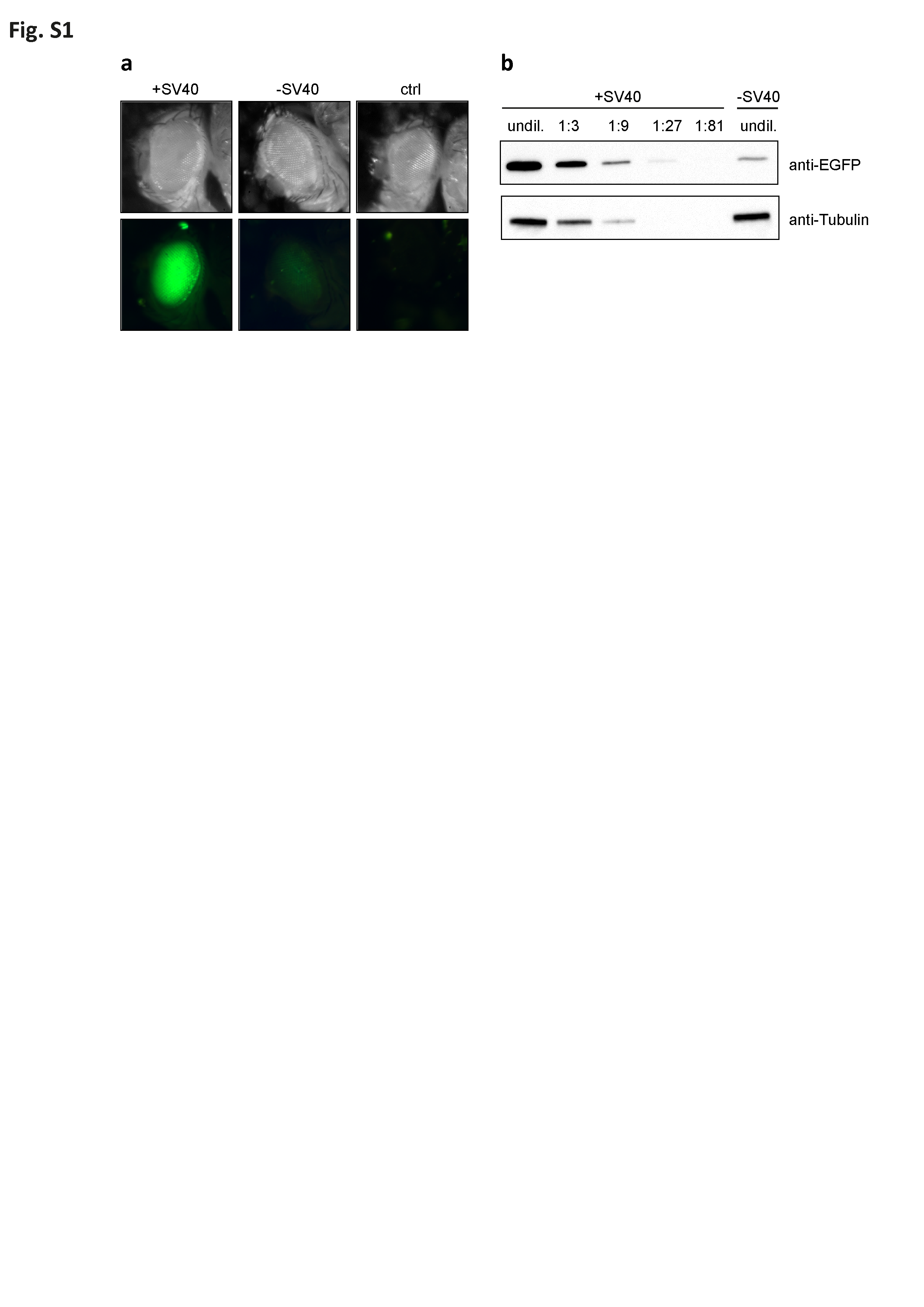

Supplement: Supplementary file 2 — High resolution image (TIF 2162 kb). [file 412_2020_733_MOESM1_ESM.tif]

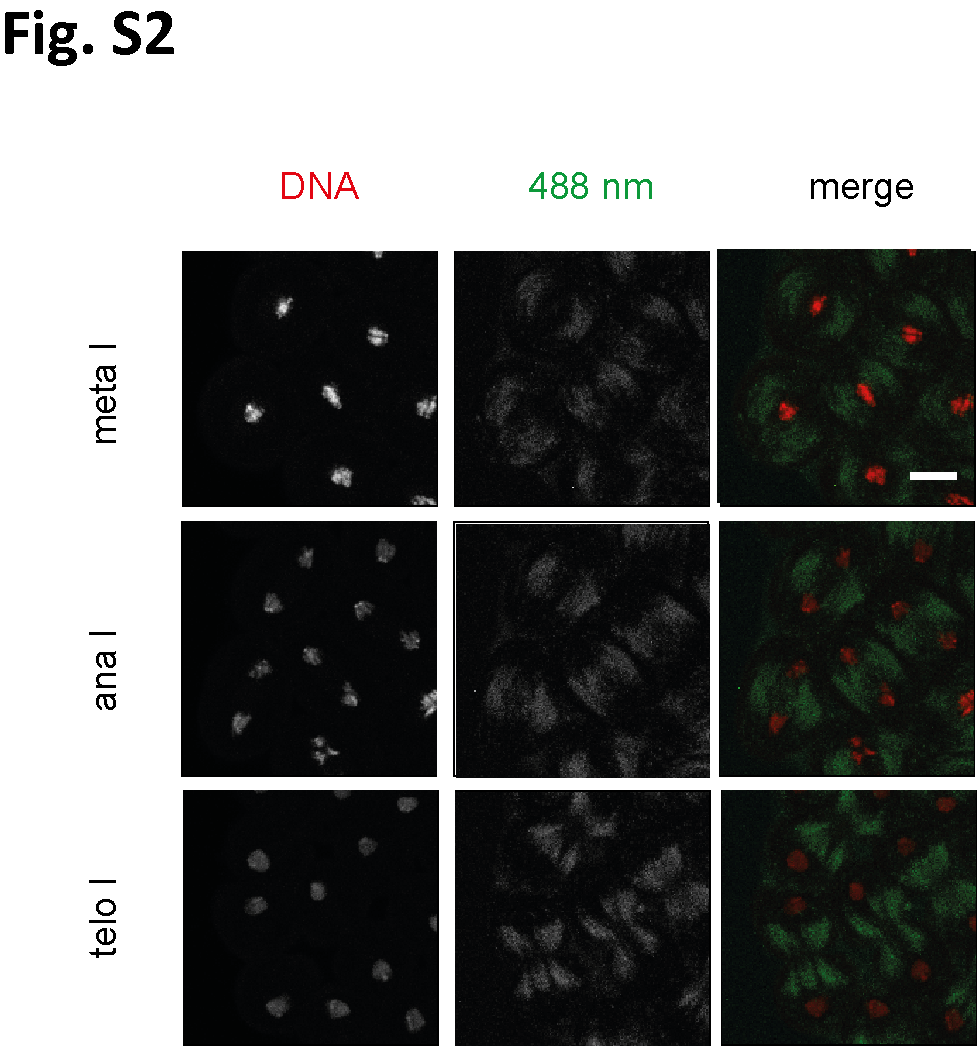

Supplement: Supplementary file 3 — Background signal in meiotic spermatocyte cysts when irradiated with the 488 nm laser line. Cysts were prepared from male pupae with the genotype Sco/CyO, P [ry+, ftz-lacZ]; His2Av-mRFP1, thus lacking any GFP-fused proteins, and observed while progressing through meiosis I. The conditions were identical to those used for generating the images shown in Fig. 2. Note the distinct cytoplasmic staining reminiscent of tubulin signals in later stages, and also the absence of any signals colocalizing with meiotic chromatin. Scale bar, 10 μm (PNG 547 kb). [file 412_2020_733_Fig7_ESM.png]

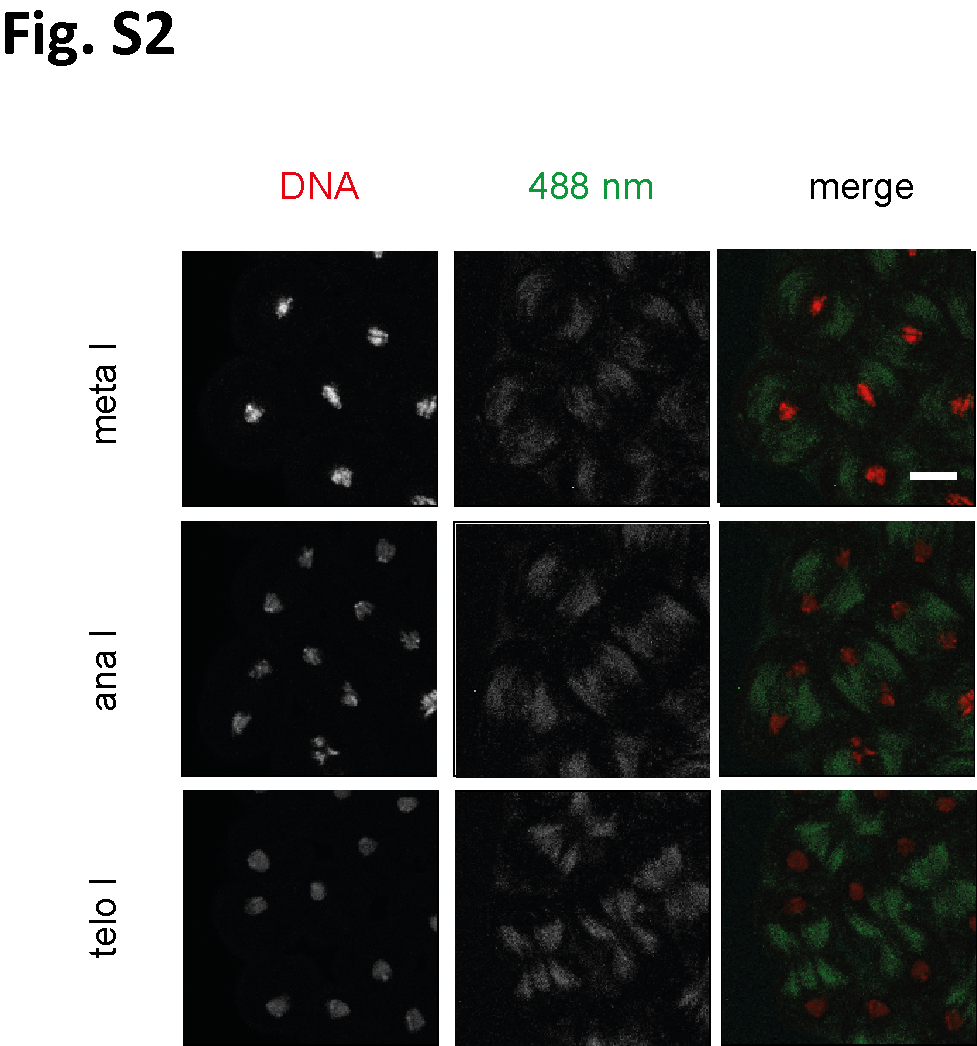

Supplement: Supplementary file 4 — High resolution image (TIF 2439 kb). [file 412_2020_733_MOESM2_ESM.tif]

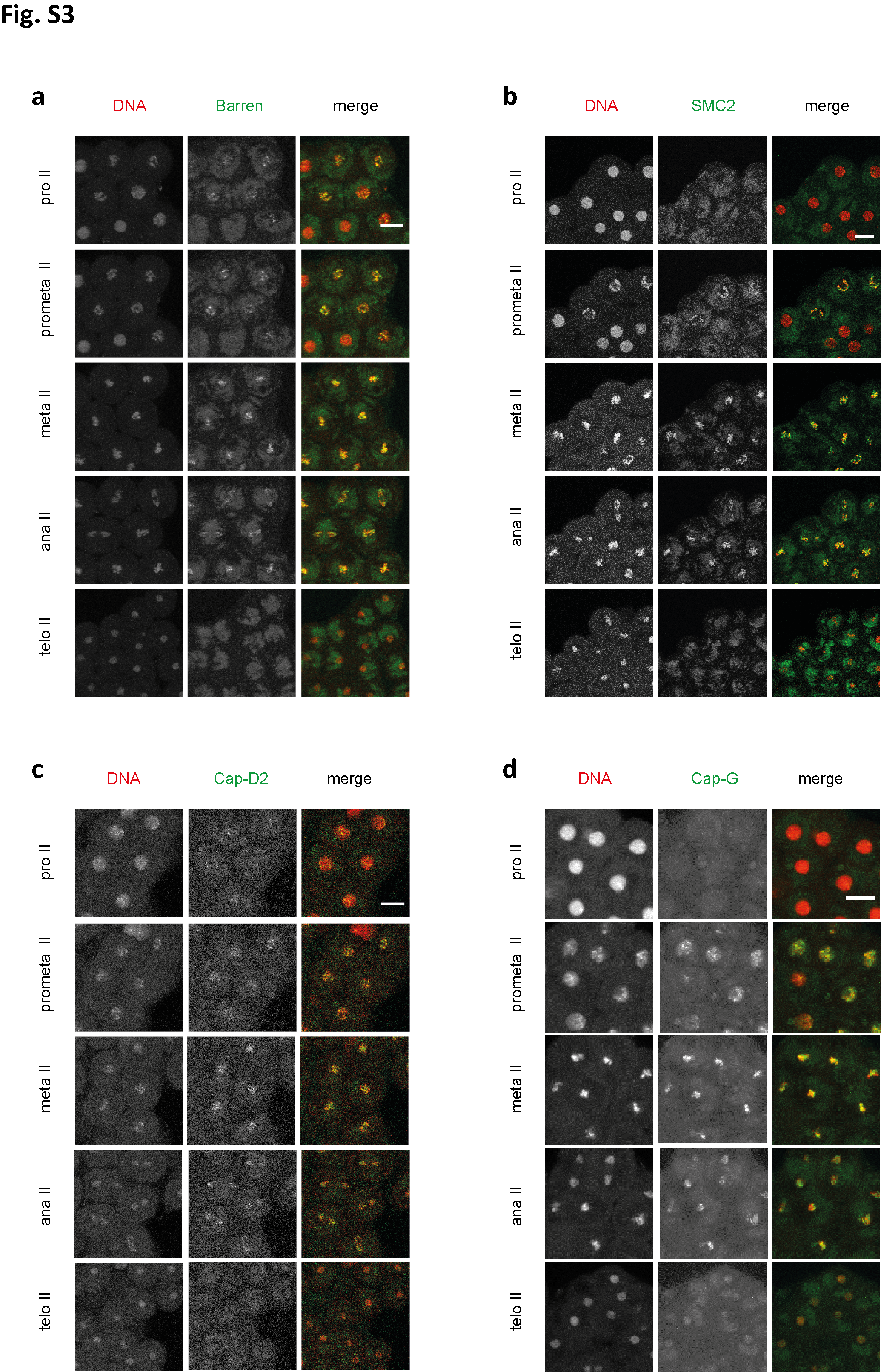

Supplement: Supplementary file 5 — Condensin I subunits localize to spermatocyte chromatin during meiosis II. Spermatocyte cysts were prepared from pupae expressing His2Av-mRFP1 to label DNA (red in merged panels) and the EGFP-fused condensin I subunits Barren (a) or SMC2 (b) or Cap-D2 (c) or Cap-G (d) (green in merged panels). These subunits were expressed in an otherwise wild-type background except for SMC2h-EGFP, which was expressed in the presence of one mutant SMC2 allele. Cysts completing meiosis I were identified. Progression through prophase (pro), prometaphase (prometa), metaphase (meta), anaphase (ana) and telophase (telo) of meiosis II was then monitored. Scale bar, 10 μm (PNG 5166 kb). [file 412_2020_733_Fig8_ESM.png]

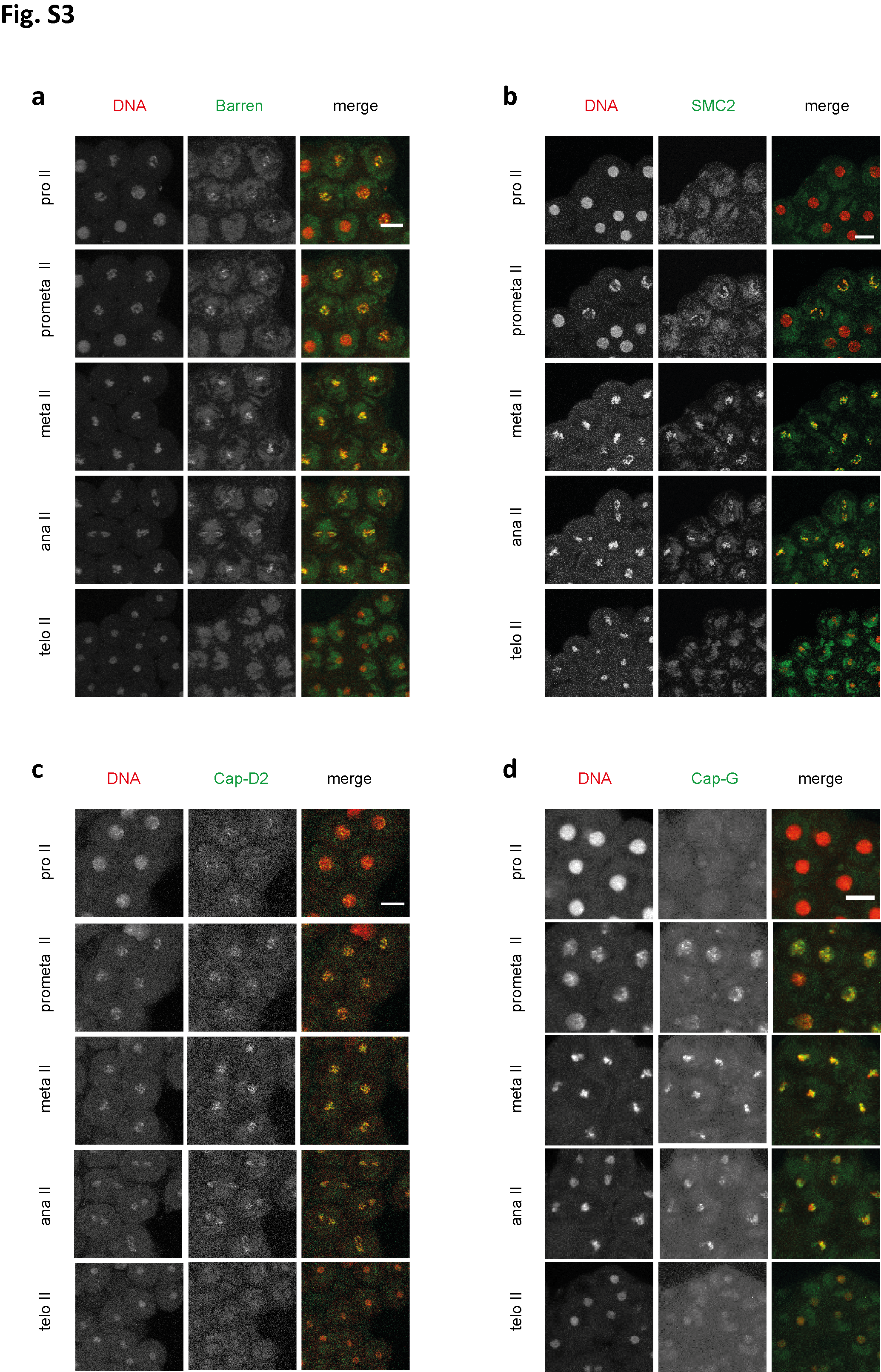

Supplement: Supplementary file 6 — High resolution image (TIF 16868 kb). [file 412_2020_733_MOESM3_ESM.tif]

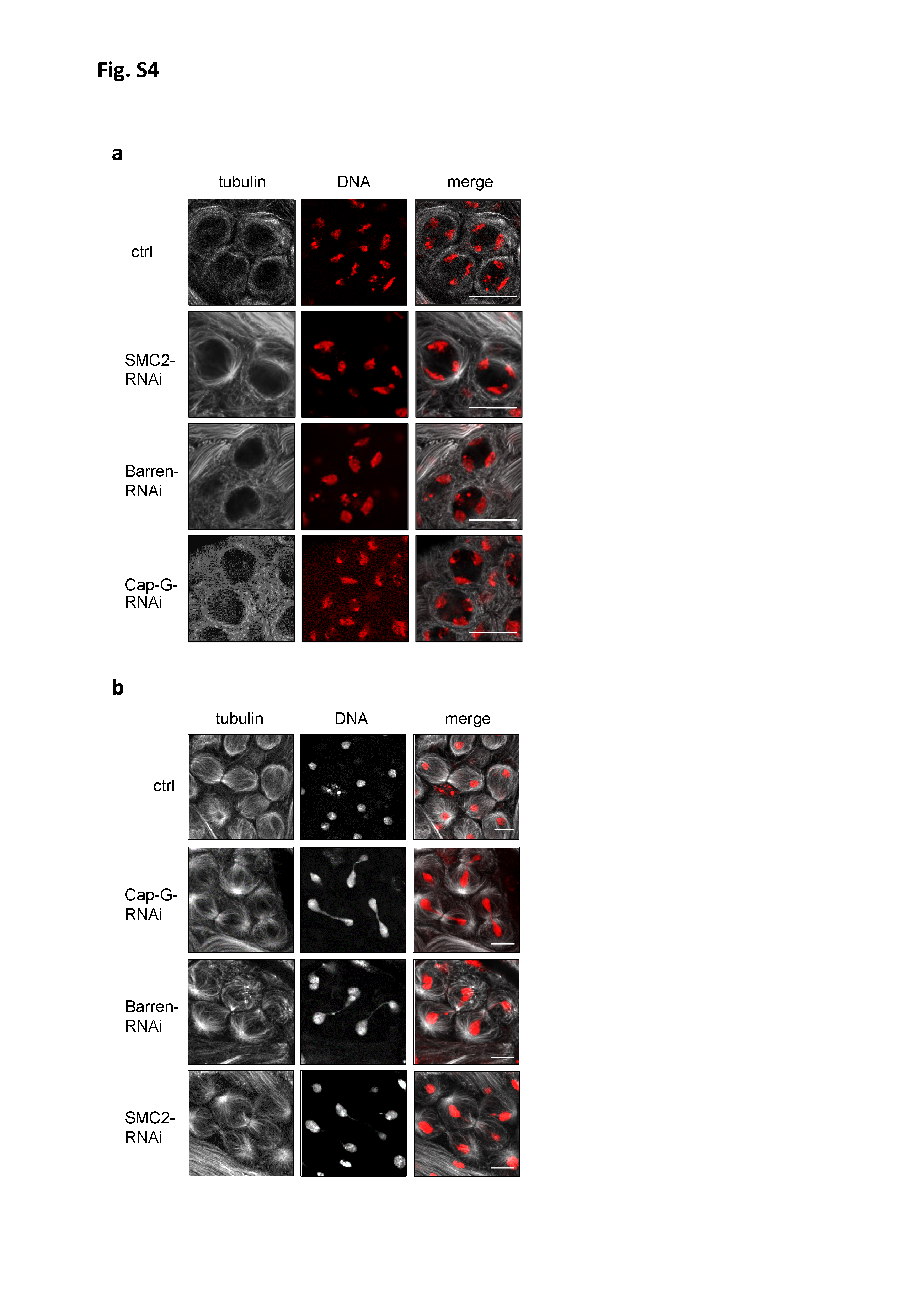

Supplement: Supplementary file 7 — Phenotypic consequences on meiosis I after depletion of condensin I subunits. a) Chromosome territories in prophase I appear normal upon condensin I depletion. b) Anaphase bridges in meiosis I are frequently present after depletion of condensin I subunits. Arrowheads highlight anaphase bridges. Testes from adult males of the genotypes w1 (ctrl), UAS-Cap-G-RNAi/bam-GAL4-VP16 (Cap-G-RNAi), UAS-Barren-RNAi/+; bam-GAL4-VP16/+ (Barren-RNAi) or UAS-SMC2-RNAi/bam-GAL4-VP16 (SMC2-RNAi) were prepared, fixed, and stained with anti-α-tubulin antibodies or Hoechst 33258 to label DNA (red in the merged panels). Scale bars, a): 25 μm; b): 10 μm (PNG 2355 kb). [file 412_2020_733_Fig9_ESM.png]

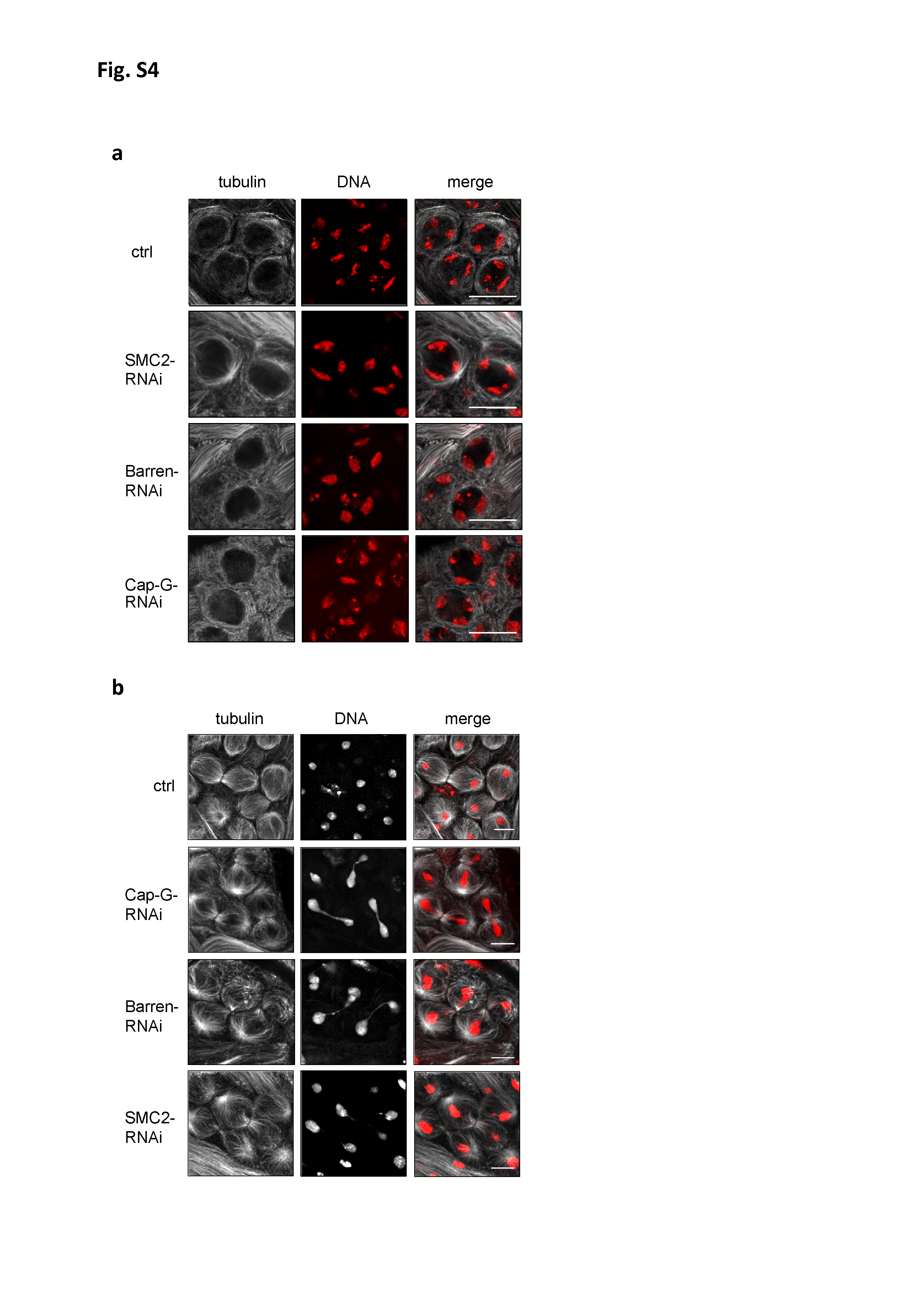

Supplement: Supplementary file 8 — High resolution image (TIF 7932 kb). [file 412_2020_733_MOESM4_ESM.tif]

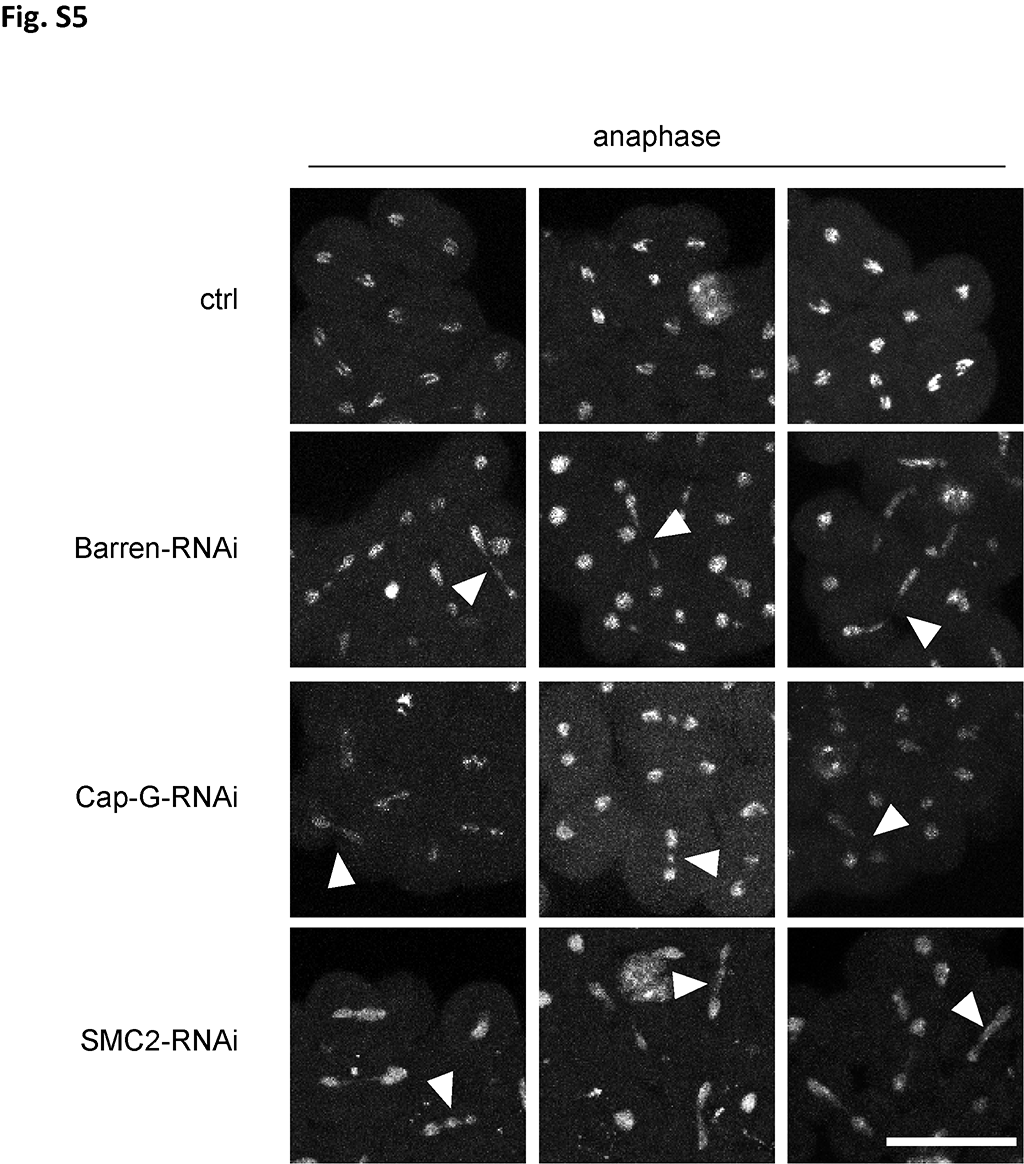

Supplement: Supplementary file 9 — Anaphase bridges in meiosis II are frequently present after depletion of condensin I subunits. Spermatocyte cysts were prepared from pupae with the genotype His2Av-mRFP1, bam-GAL4-VP16 (ctrl) or UAS-Cap-G-RNAi/His2Av-mRFP1, bam-GAL4-VP16 (Cap-G-RNAi) or UAS-Barren-RNAi/+; His2Av-mRFP1, bam-GAL4-VP16/+ (Barren-RNAi), or UAS-SMC2-RNAi/His2Av-mRFP1, bam-GAL4-VP16 (SMC2-RNAi). Cysts entering meiosis II were identified by the number and the size of the nuclei within the cysts, as revealed by chromatin-associated His2Av-mRFP1. Progression through meiosis II was monitored by in vivo microscopy. Three examples for anaphase figures of each genotype are shown. Arrowheads indicate examples of anaphase bridges. Scale bar, 25 μm. (PNG 3.41 mb). [file 412_2020_733_Fig10_ESM.png]

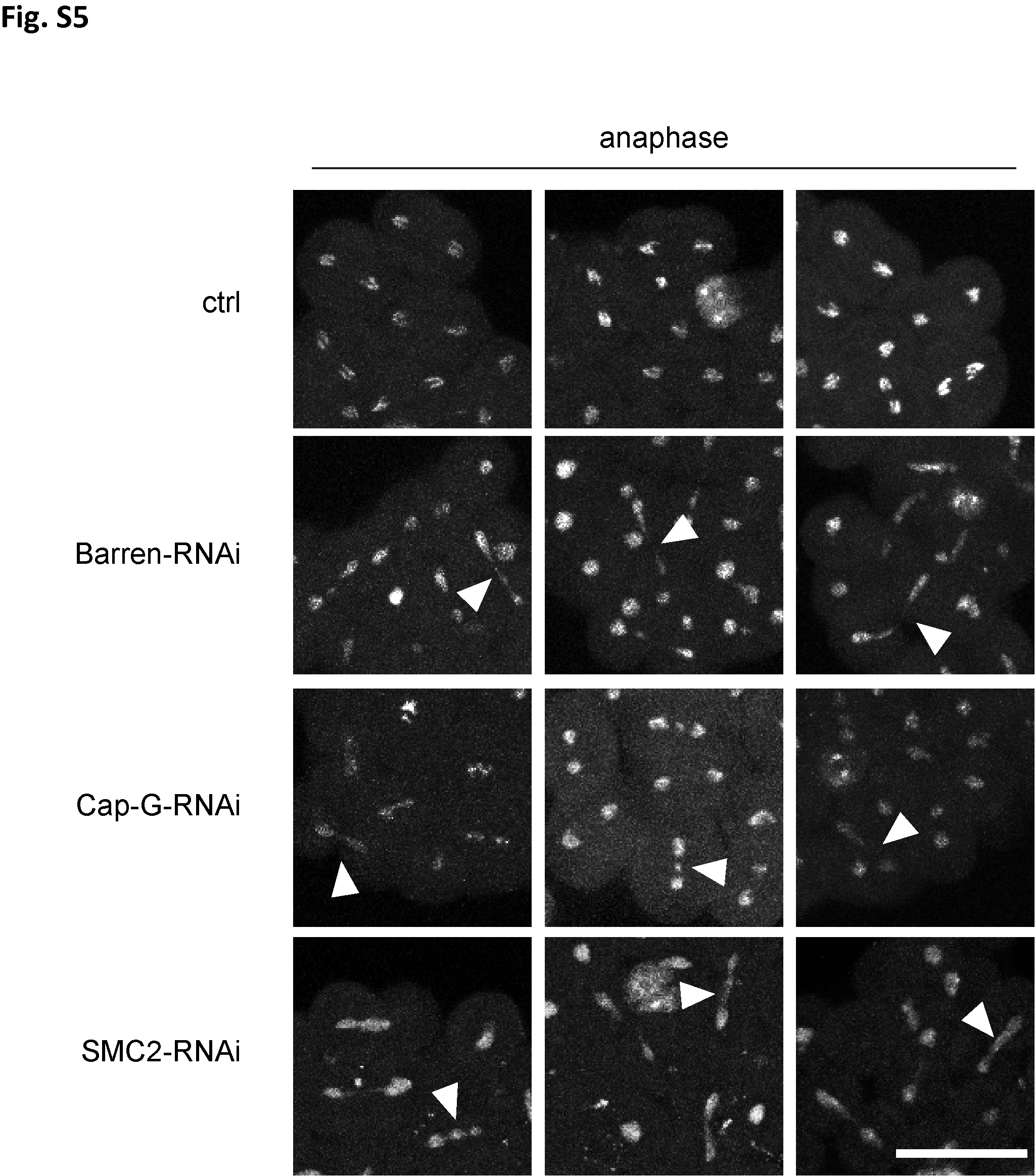

Supplement: Supplementary file 10 — High resolution image (TIF 6.56 mb). [file 412_2020_733_MOESM5_ESM.tif]

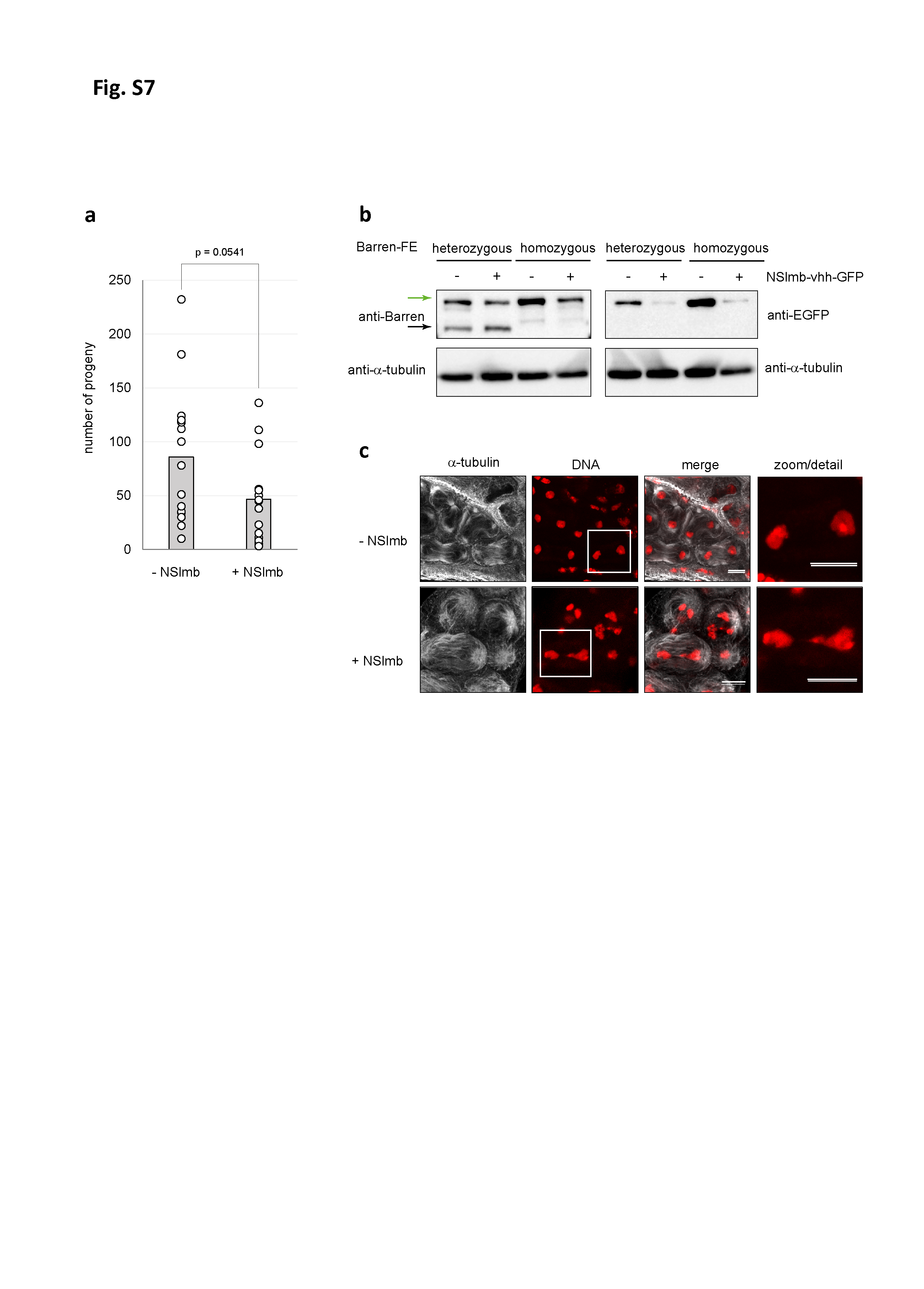

Supplement: Supplementary file 12 — Condensin I depletion via induced proteasomal degradation results in reduced male fertility and chromatin bridges during meiosis. a) Individual males of the genotypes Barren-FE; UASP-NSlmb-vhh4-GFP/TM3, Sb (-NSlmb) or Barren-FE; UASP-NSlmb-vhh4-GFP/bam-GAL4-VP16 (+NSlmb), were mated with w1-virigins, and the number of progeny was counted. b) Western Blot analysis of fly strains expressing Barren-FE exclusively (homozygous), or in the presence of one wild-type allele (heterozygous) in the presence (+) or absence (−) of NSlmb-vhh4-GFP. The green and the black arrow indicate the EGFP-fusion products and the endogenous proteins, respectively. c) Testes from adult males of the genotypes Barren-FE; UASP-NSlmb-vhh4-GFP/TM3, Sb (-NSlmb) or Barren-FE; UASP-NSlmb-vhh4-GFP/bam-GAL4-VP16 (+NSlmb) were prepared, fixed, and stained with anti-α-tubulin antibodies or Hoechst 33258 to label DNA (red in the merged panels). Anaphase bridges were more frequently observed in the presence of NSlmb-vhh4-GFP (5 out of 13 anaphase cells) when compared to the absence of NSlmb-vhh4-GFP (1 out of 18 cells). Examples for anaphase figures are shown in the enlarged panels on the right. Scale bars, 10 μm (PNG 718 kb). [file 412_2020_733_Fig11_ESM.png]

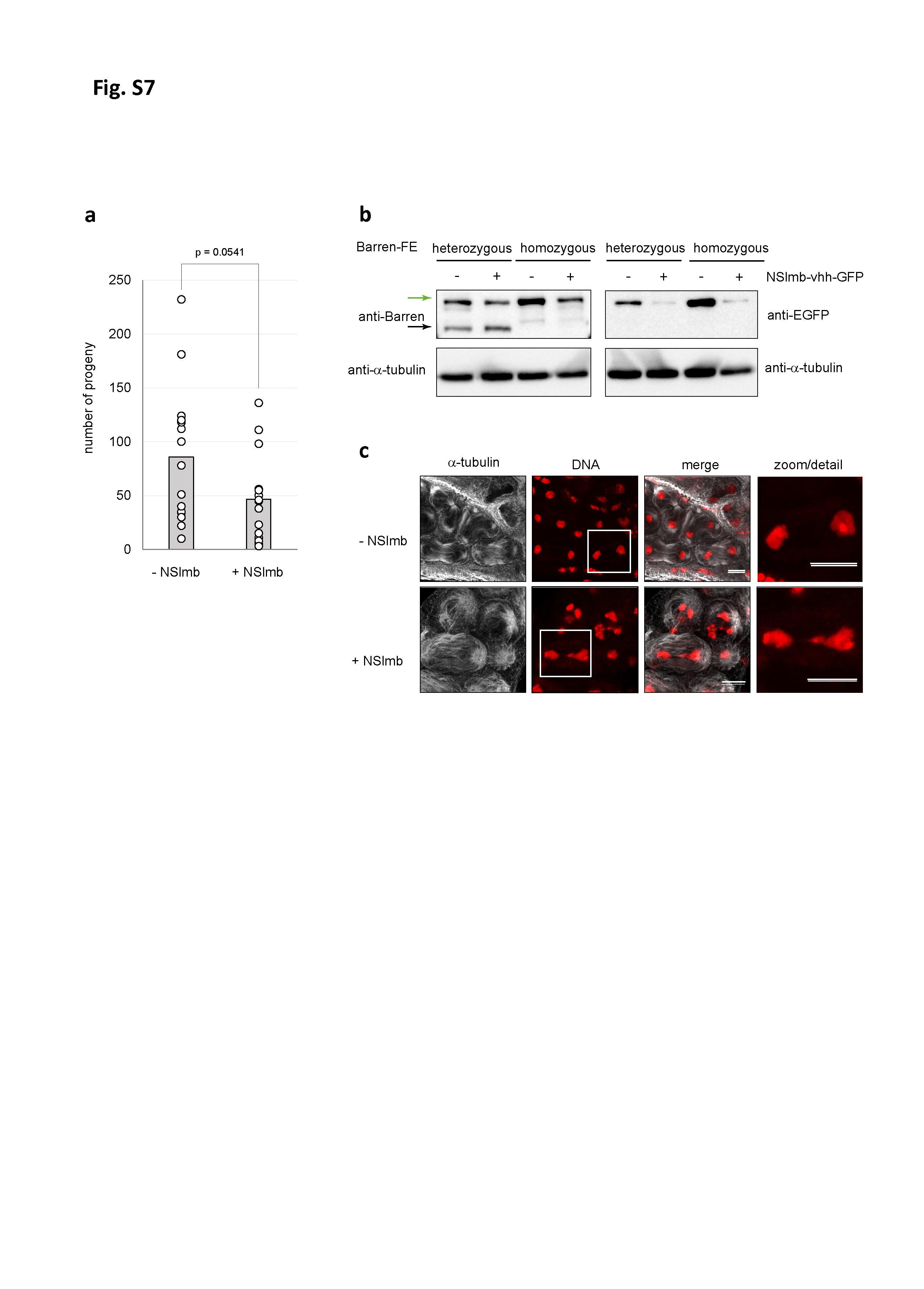

Supplement: Supplementary file 13 — High resolution image (TIF 3440 kb). [file 412_2020_733_MOESM7_ESM.tif]
