# Supplementary material for: Condensin I is required for faithful meiosis in Drosophila males
Source: Chromosoma. 2020 Apr 8;129(2):141–60. doi: 10.1007/s00412-020-00733-w (PMC7260282; doi:10.1007/s00412-020-00733-w)
Supplement: Supplementary file 11 — Specificity of phenotypes caused in mitotic proliferating tissue by RNAi-induced downregulation of condensin I function. a) Schematic representation of the final cross to assess suppression of RNAi-induced phenotypes. Condensin-EGFP is an abbreviation for either a wild-type (RNAi-sensitive) or RNAi-resistant transgene variant of the three analyzed condensin genes. The transgenes are expressed under control of the genomic flanking sequences, and they are all inserted at the same genomic position on the third chromosome (68E) via the φC31 integrase system. UAS-Condensin-siRNA represents transgenes expressing double-stranded RNAs under UAS control targeting SMC2, Cap-G or Barren. These transgenes are located on either chromosome II (Barren) or chromosome III (SMC2, Cap-G). b) Schematic representation of the progeny classes expected from the cross shown in a). The various chromosome combinations result in expression of neither siRNA nor condensin-EGFP (class I), expression of only condensin-EGFP (class II), expression of only siRNA (class III) or expression of both siRNA and condensin-EGFP (class IV, green shading). c) Assignments of the individuals resulting from the crosses to the various progeny classes. The expression of the siRNAs under control of the ey-GAL4 driver result in complete (SMC2 and Cap-G) or almost complete (Barren) lethality, when only endogenous condensin is expressed (class III). Transgenic expression of only the RNAi-resistant variants significantly rescues this lethality (class IV). The few individuals which eclosed in the absence of an RNAi-resistant transgene in the case of Barren, point to an incomplete destruction of the corresponding mRNA. However, these individuals were all characterized by severe malformations of the eyes, which was observed in only the minority of the cases, when the RNAi-resistant transgene was expressed. (DOCX 14 kb). [file 412_2020_733_MOESM6_ESM.docx]

**Fig. S6**

**a**

Final cross: ♀♀ $\frac{ey-GAL4}{CyO}$ ; $\frac{Condensin-EGFP}{TM3}$ x ♂♂ $\frac{UAS-Condensin-siRNA}{UAS-Condensin-siRNA}$

**b**

| Progeny class | Chromosomes inherited from mothers | | Transgenic chromosome inherited from fathers | Expression | |
| --- | --- | --- | --- | --- | --- |
|  | Chromosome II | Chromosome III | Chromosome II or III | siRNA | Condensin-EGFP |
| I | *CyO* | *TM3* | *UAS-Condensin-siRNA* | - | - |
| II | *CyO* | *Condensin-EGFP* | *UAS-Condensin-siRNA* | - | + |
| III | *ey-GAL4* | *TM3* | *UAS-Condensin-siRNA* | + | - |
| IV | *ey-GAL4* | *Condensin-EGFP* | *UAS-Condensin-siRNA* | + | + |

**c**

| Progeny class | *SMC2_h_-EGFP* | | *Cap-G-EGFP* | | *Barren-EGFP* | |
| --- | --- | --- | --- | --- | --- | --- |
|  | wild-type | RNAi-resistant | wild-type | RNAi-resistant | wild-type | RNAi-resistant |
| I | 22 (42%) | 54 (38%) | 36 (51%) | 39 (27%) | 97 (46%) | 107 (28%) |
| II | 31 (58%) | 51 (35%) | 34 (49%) | 54 (38%) | 108 (51%) | 103 (27%) |
| III | - | - | - | - | 1 (0.5%) | 1 (0.3%) |
| IV | - | 39 (27%) | - | 49 (35%) | 5 (2.5%) | 165 (44%) |
